# The role of relaxins in blood cell modulation: interactions with relaxin family peptide receptor 1 (RXFP1) and glucocorticoid receptor (GR)

**DOI:** 10.1042/CS20256619

**Published:** 2025-11-06

**Authors:** Weronika Broszkiewicz, Kamila Domińska

**Affiliations:** Department of Comparative Endocrinology, Medical University of Lodz, Lodz, Zeligowskiego 7/9, 90-752, Poland

**Keywords:** blood cells, cytokines, glucocorticoid receptor, immunomodulation, relaxin, RXFP1

## Abstract

The relaxin family functions as pleiotropic hormones with various antioxidant, angiogenic, anti-apoptotic, anti-hypertrophic, anti-inflammatory, antifibrotic, and vasodilatory effects. To fully appreciate the potential therapeutic applications of relaxins and the pathophysiological implications, it is important to understand their multifaceted roles. This comprehensive review of current literature aims to elucidate the role of relaxins in modulating the biology and function of blood cells. It places special emphasis on the signaling pathways of relaxin family peptide receptor 1 (RXFP1) and the glucocorticoid receptor (GR) activated by relaxin-2. Relaxin-2 influences circulating blood cell counts and exerts inhibitory effects on megakaryocytes, thrombocytes, and mast cells. It also possesses immunomodulatory characteristics that affect granulocytes and agranulocytes, particularly regarding their morphology, differentiation, and function. Relaxin-1 regulates dendritic cell maturation and cytokine secretion. RXFP1 could play significant roles in blood malignancies and preeclampsia. The broad spectrum of activities demonstrated by relaxins significantly influences blood cell biology and highlights their therapeutic potential in a range of conditions, including hematological, cardiovascular, renal, pregnancy-related, and fibrotic disorders.

## Introduction

Relaxin-2 is one of the better-known members of the insulin superfamily. Originally characterized as a peptide hormone involved in the relaxation of the interpubic ligament during pregnancy [1,2], its biological roles are now recognized to extend far beyond connective tissue remodeling, pregnancy, and female physiology [1,3]. However, in humans, measurable circulating relaxin-2 is generally found only during pregnancy. Previous reports suggesting its presence in males or nonpregnant females likely reflect limitations of earlier, unvalidated assays [4,5]; indeed, more recent studies using rigorous approaches, including immunoaffinity mass spectrometry, confirm that systemic relaxin-2 levels outside pregnancy are minimal or absent [6]. Nevertheless, relaxin-2 is also secreted locally by different tissues of nonpregnant women and men, and it is now described as a remarkable pleiotropic hormone in the human body, inducing a wide range of actions far beyond reproduction [7]. Importantly, its effects have been observed not only in various organs and tissues but also at the level of individual blood cell populations. There is growing evidence that relaxin affects the numbers of morphotic elements, as well as their differentiation, morphology, and function. This literature review will focus on the effect of relaxins on individual blood cells, such as megakaryocytes, mastocytes, granulocytes, and agranulocytes, detailing the specific signaling pathways believed to be associated with its action in both physiological and pathological states.

Relaxin-2 temporarily influences the quantity of specific circulating cellular blood components, including an elevation in white blood cell count, a decrease in lymphocyte numbers and a significant increase in neutrophil numbers, and a reduction in red blood cell count [8]. Relaxin-2 has also been demonstrated to exert significant effects on blood cell biology, such as the modulation of immune cell function, as well as platelet aggregation, and neutrophil, mast cell, and T cell activity. It also influences immune cell recruitment and polarization, with potential implications in inflammation and hematological disorders. Relaxin signaling pathways and its receptors may represent promising therapeutic targets for treating various immune-related conditions, such as rheumatoid arthritis and blood neoplasms.

## Relaxins: versatile peptide hormones and their receptors

The relaxin peptide family consists of seven members classified into the relaxin isoforms encoded by the *RLN1-3* genes, and the insulin-like peptides encoded by *INSL3-6* [9]. Mature relaxin is a two-chain (A and B) peptide, with a molecular weight of approximately 6 kDa. It contains one intrachain (Cys10′-Cys15′) and two interchain (Cys10-Cys11′ and Cys22-Cys24′) disulfide bonds [10]. In most of the literature, the term ‘relaxin’ typically refers to the human *RLN2* gene product. In lower species, however, the corresponding ortholog is referred to as *Rln1*, reflecting differences in nomenclature across species [11]. As a hormone, relaxin demonstrates pleiotropic potential ranging from antioxidant, anti-inflammatory, and anti-apoptotic effects, to anti-hypertrophic, angiogenic, anti-fibrotic, and vasodilatory properties [12]. These wide-ranging effects arise through its interaction with other peptides and proteins. In 2002, two cognate receptors for relaxin-2 were identified, both classified as G protein-coupled receptors, namely LGR7 (now RXFP1) and LGR8 (now RXFP2), and shortly thereafter in 2003, relaxin-3 was discovered as a ligand for two additional receptors, GPCR135 (now RXFP3) and GPCR142 (now RXFP4) [9,13–15]. Among these, RXFP1 is regarded as the primary and physiologically relevant receptor for RLN-2, whereas there is still no evidence that RLN-2 induces physiological activation of RXFP2 *in vivo* [16]. It was also reported that it may be a functional endothelin-1 antagonist [17]. However, surprisingly, relaxin-2 was later identified as a glucocorticoid receptor (GR) agonist [18], while cortisol is the main physiological GR ligand that modulates immune activity [19]. This observation, particularly in light of the fact that GR is expressed in nearly all human cells [20], provides a new perspective on the potential immunomodulatory role of relaxin-2.

### RXFP1: the primary receptor mediating relaxin signaling

**Figure 1:**
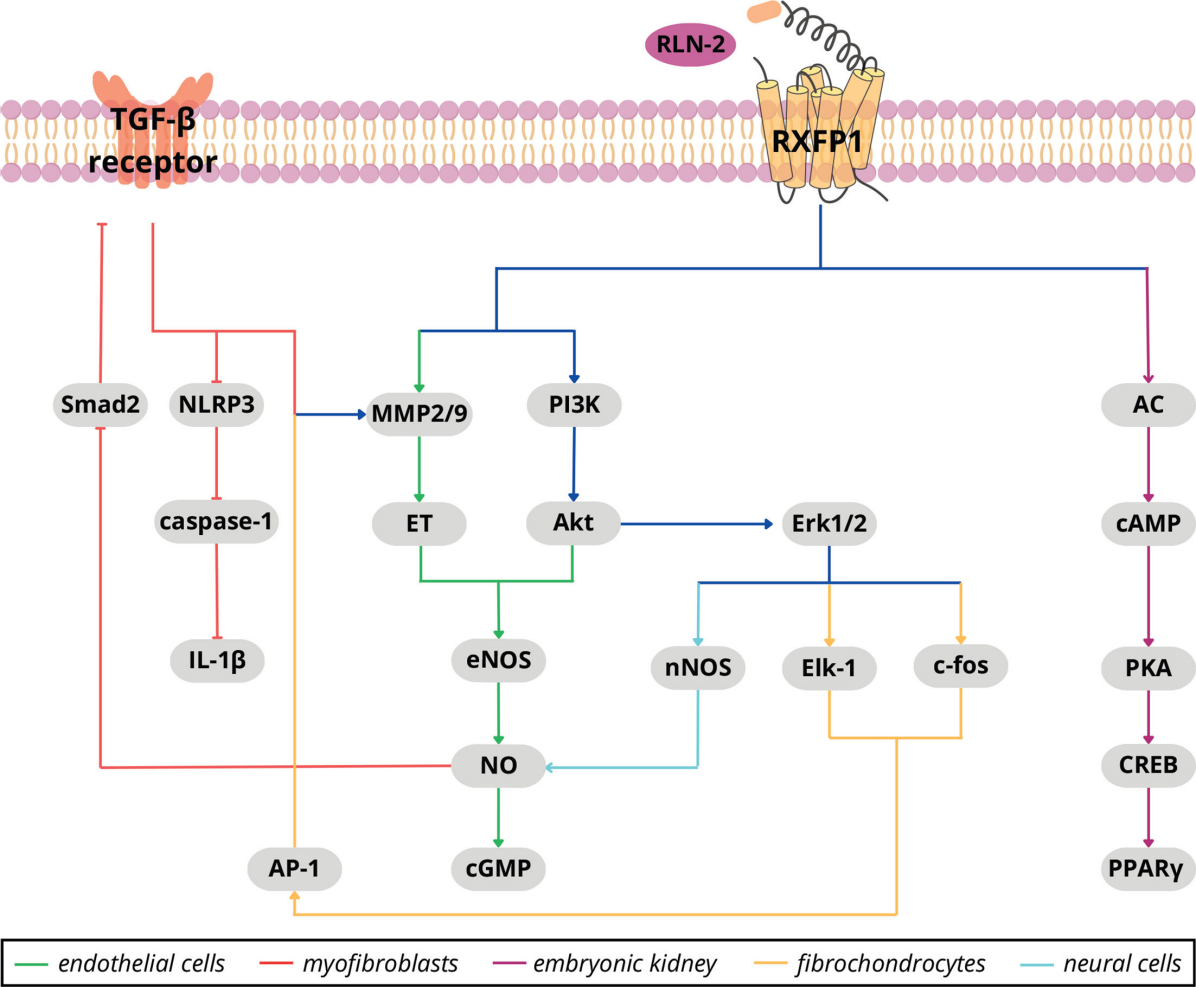
Schematic representation of RXFP1 signaling in a cell-type–dependent context. ↑ activation, ^┬^ inhibition. AC, adenylyl cyclase; Akt, protein kinase B; AP-1, activator protein 1; CREB, cAMP response element–binding protein; eNOS, endothelial nitric oxide synthase; Elk-1, ETS-like transcription factor 1; Erk1/2, extracellular signal-regulated kinases 1/2; ET, endothelin; IL-1β, interleukin-1 beta; MMP2, MMP9, matrix metalloproteinases 2 and 9; NLRP3, NOD-like receptor family pyrin domain–containing 3; nNOS, neuronal nitric oxide synthase; PI3K, phosphoinositide 3-kinase; PKA, protein kinase A; PPARγ, peroxisome proliferator–activated receptor gamma; Smad2, SMAD family member 2; TGF-β, transforming growth factor beta.

RXFP1 is a class A G protein–coupled receptor with a unique extracellular architecture: it contains an N-terminal low-density lipoprotein class A (LDLa) module tethered to a leucine-rich repeat (LRR) domain, both of which are essential for ligand recognition and receptor activation [21]. Binding of relaxin to the LRR domain promotes intramolecular interactions between the LDLa module and the transmembrane bundle, thereby stabilizing an active GPCR conformation and enabling coupling to multiple G proteins [22]. Canonical RXFP1 activation stimulates G_s_ to increase cAMP/PKA signaling, but the receptor can also engage G_i/o_, PI3K/Akt, ERK1/2, PKC-ζ, and NO–sGC–cGMP pathways, with the balance of these outputs depending strongly on the cell type (Figure 1).

At the vascular interface, endothelial RXFP1 couples via PI3K–Akt to eNOS, boosting NO and cGMP to drive vasodilation and anti-inflammatory tone, with additional remodeling signals through MMPs and endothelin/ET_B_ cross-talk [23]. In mesenchymal compartments, RXFP1 activation in dermal, cardiac, and renal fibroblasts/myofibroblasts triggers ERK1/2 and nNOS-derived NO, up-regulates collagen-degrading MMPs, and interrupts TGF-β/Smad2 signaling to reverse myofibroblast differentiation [24–26]. Analogous RXFP1-dependent anti-fibrotic signaling is seen in hepatic stellate cells, where the receptor dampens TGF-β/Smad activity, enhances collagenase expression, and can engage transcriptional effectors such as PPARγ [27,28]. In cartilage-like fibrochondrocytes, RXFP1 drives ERK/PI3K pathways to induce MMP-9/13 and remodel extracellular matrix, highlighting a catabolic program distinct from the anti-fibrotic response in classic fibroblasts [29]. Within reproductive tissues, myometrial and endometrial signaling through RXFP1 prominently elevates cAMP and ERK activity and interfaces with steroid pathways [30]. Finally, in neural cells, RXFP1 engagement can activate ERK–nNOS–NO signaling and confer protection against oxidative stress and apoptosis, underscoring the receptor’s breadth beyond connective and vascular biology [31].

The relaxin hormone peptide family may also play a significant role in various malignancies, including leukemia. The influence of relaxin on leukemia is slowly becoming an important area of cancer research, particularly due to the significance of its receptor RXFP1. RXFP1 expression is markedly elevated in leukemia cells, with 2.71-fold increases in acute myeloid leukemia (AML) and 3.31-fold increases in chronic myeloid leukemia (CML) compared with control populations (CD34^+^/CD38- stem cells and CD34^+^/CD38^+^ progenitor cells) [32]. RXFP1 expression is notably higher in chronic leukemias compared with acute forms, hinting at its potential involvement in disease progression. Interestingly, the literature also suggests a correlation between high RXFP1 expression and poorer cancer-specific survival rates in leukemia patients, especially those with mutations in the RXFP1 gene [32]. This association raises important questions about the clinical implications of RXFP1 in leukemia treatment and prognosis.

### The GR as an alternative mediator of relaxin action

The concept that relaxin-2 may function as a GR agonist emerged following the observation that RU486, a GR antagonist, abolished the immunosuppressive effects of both dexamethasone (DEX) and RLN-2 on pro-inflammatory cytokine secretion (IL-1, IL-6, and TNF-α) in THP-1 cells [33]. This prompted Dschietzig et al. to investigate the interaction further, ultimately demonstrating that RLN-2 activates GR and induces expression of genes under glucocorticoid response element (GRE) control. RLN-2 not only suppressed cytokine secretion more potently than DEX, despite DEX having a greater maximal effect, but also promoted GR nuclear translocation and transcriptional activity in a manner consistent with classical GR agonists. Additionally, it was proposed that this interaction can subsequently enhance expression and function of other steroid receptors, such as androgen receptor [34].

Mechanistically, RLN-2 was shown to associate with the GR complex under both basal and stimulated conditions, as evidenced by co-immunoprecipitation of RLN-2 with GR and its cytoplasmic chaperones Hsp70 and Hsp90 [33]. Upon stimulation, GR-RLN-2 complexes translocated to the nucleus, with peak nuclear accumulation observed within 30 min—comparable with DEX. This translocation was further visualized via fluorescence microscopy, which revealed concentration-dependent intracellular uptake and nuclear localization of RLN-2. In direct binding assays, RLN-2 displayed high-affinity binding to GR (IC50~0.4–4 nM), outperforming DEX and corticosterone (IC50~12 nM and 115 nM, respectively), and induced phosphorylation of serine 211 on GR, a hallmark of receptor activation.

Functionally, RLN-2 mirrored DEX in modulating GR-dependent anti-inflammatory pathways, including up-regulation of antioxidant enzymes and inhibition of NF-κB and AP-1 transcriptional activity. These effects were abolished by RU486 or GR gene silencing, confirming GR dependency [35]. *In vivo*, RLN-2 ameliorated TNFα-induced endothelial dysfunction and acute pancreatitis in rodent models by reducing pro-inflammatory cytokines—effects again reversed by RU486, indicating GR involvement without excluding a role for RXFP1 [36].

Importantly, RLN-2 retained its GR-binding capacity even when chemically modified to prevent RXFP1 interaction, suggesting that GR activation can occur independently of classical relaxin receptor [37]. Both native and RXFP1-inactive RLN-2 analogs displaced fluorescent glucocorticoid tracers from GR (IC50~4–6 nM) and blunted endotoxin-induced TNF-α and IL-6 secretion in THP-1 cells—a response blocked by both non-selective (RU486) and selective (D06) GR inhibitors. Notably, inhibition of the MEK1/2–ERK1/2 pathway had no effect, supporting the conclusion that RLN-2’s immunomodulatory function operates through GR rather than MAPK signaling. However, Brecht et al. reported that RU486 did not affect stimulated chemokine expression in HUVEC or THP-1 cells [38]. Importantly, ERK-1/2, Akt, and ET_B_ signaling activated by RLN-2 via RXFP1 seem to occur independently of GR signaling [37].

Moreover, RLN-2 significantly enhanced GR expression at both the mRNA and protein levels [18]. GR-α mRNA increased within 30 min to 4 h in HeLa, THP-1, and 293 cells, while GR-β mRNA rose in HeLa and 293 cells. At the protein level, GR levels increased up to ~500% of baseline in HeLa and ~280% in THP-1 cells—effects abolished by RU486. Promoter assays and chromatin immunoprecipitation demonstrated that RLN-2 not only activated its own gene promoter via GR-dependent mechanisms but also directly enhanced GR binding to GRE half-sites within the relaxin-2 promoter in HeLa and THP-1 cells [39]. These findings were reinforced by immunofluorescence data showing nuclear co-localization of GR and RLN-2, a phenomenon not observed with insulin, and by experiments in GR-deficient HT-29 cells, where transfection of GR restored RLN-2 responsiveness.

Crucially, studies in spleen fibroblasts—cells lacking both RXFP1 and RXFP2 (confirmed by PCR)—demonstrated preserved RLN-2-induced GRE-luciferase activity and GR up-regulation, further supporting GR activation in an RXFP1-independent context [33]. Furthermore, the anti-inflammatory effects of RLN-2 on decidual macrophages, which do not express the progesterone receptor, are thought to be mediated primarily via GR rather than RXFP1, as indicated by their reversal upon treatment with RU486 [40]. Conversely, in HEK293T cells, which also lack RXFP1/2, Michelle et al. found that RLN-2, RLN-3, and INSL3 did not activate GRE [41]. However, RLN-2 triggered a GRE reporter response in RXFP1-transfected HEK293T cells—but not in RXFP2-transfected ones—and this effect was independent of G_i_/G_o_ protein coupling, suggesting possible cell type—or receptor-context specificity. Reports by Michelle et al. [41] and others [42], describing absent RXFP1/2 expression in HEK293 cells are contradicted by earlier studies showing their presence [33], underscoring discrepancies across studies. Differences in experimental design—including cell type (spleen fibroblasts vs. HEK293T), reporter systems (luciferase vs. secreted alkaline phosphatase), and RLN-2 concentrations (0–20 μM vs. 100 μM)—are likely to account for the conflicting results regarding dependence on RXFP1. Horton et al. suggested that RLN-2 receptor engagement may be dose-dependent, with lower concentrations preferentially activating GR and higher concentrations activating RXFP1, providing a potential explanation for the observed discrepancies [40]. Relaxin-2 may also use different amino acid residues to bind and activate RXFP1 and GRs, contributing to its diverse biological effects on immunity, cardiovascular function, and connective tissue [37]. While RXFP2 can be excluded from involvement in GR activation, the necessity of RXFP1 remains unresolved and may vary by context. Overall, the current body of evidence robustly supports RLN-2 as a functional GR agonist, but further investigation is required to clarify the contribution of RXFP1 and to delineate the concentration-dependent dynamics of receptor specificity.

## Relaxins and their impact on blood cell function

**Figure 2:**
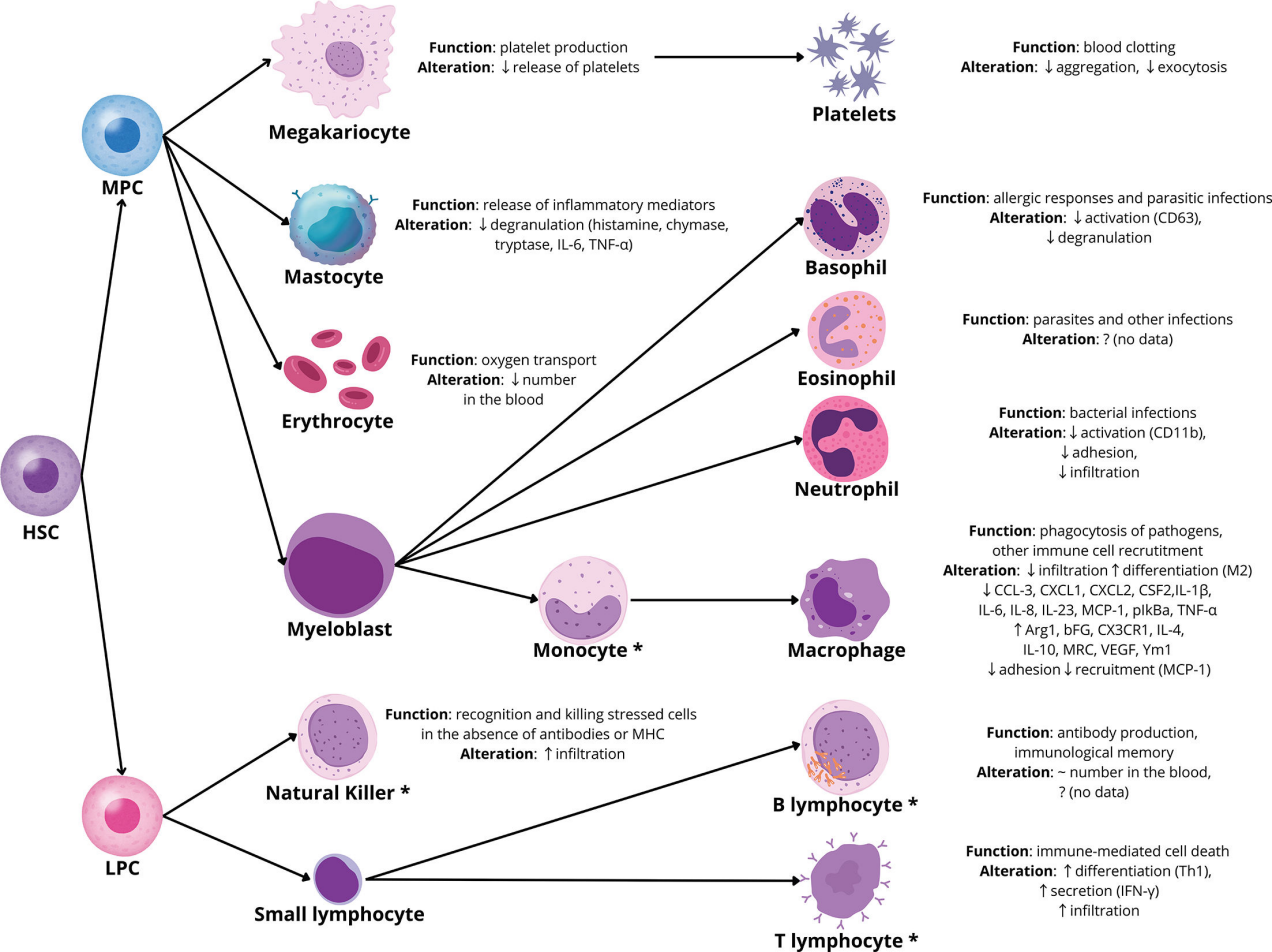
The impact of relaxin-2 on the biology of morphotic elements of the blood. ↓ down-regulation, ↑ up-regulation, ~ no change, ? no data, * belonging to PBMC. Arg1, arginase 1; bFGF, basic fibroblast growth factor; CCL-3, chemokine (C-C motif) ligand 3; CD63, cluster of differentiation 63; CSF2, colony stimulating factor 2; CX3CR1, C-X3-C motif chemokine receptor 1; CXCL1, C-X-C motif chemokine ligand 1; CXCL2, C-X-C motif chemokine ligand 2; HSC, hematopoietic stem cell; IFN-γ, interferon gamma; IL-1, interleukin 1; IL-1β, interleukin 1 beta; IL-10, interleukin 10; IL-23, interleukin 23; IL-4, interleukin 4; IL-6, interleukin 6; IL-8, interleukin 8; LPC, lymphoid progenitor cell; MCP-1, monocyte chemoattractant protein 1; MRC, mannose receptor C-type; MPC, multipotent progenitor cell; pIkBa, phosphorylated inhibitor of kappa B alpha; TNF-α, tumor necrosis factor alpha; VEGF, vascular endothelial growth factor; Ym1, Ym1 protein, a chitinase-like protein.

Blood cells are classified by function and origin, all deriving from hematopoietic stem cells (HSCs). HSCs first become progenitor cells, then differentiate into blood cell types. Myeloid progenitors form erythroid and myeloid lineages, while lymphoid progenitors generate lymphoid cells [43]. A simplified overview of blood cell formation and the impact of relaxin-2 on individual blood morphological elements is illustrated below (Figure 2). In the subsequent sections of the article, the effects of relaxin on selected blood cell types, including megakaryocytes, mast cells, granulocytes, and agranulocytes, are discussed.

## The effect of relaxin-2 on megakaryocytes and platelets

Megakaryocytes, large bone marrow cells, and their derivatives, platelets, play a central role in hemostasis but also function as immune sentinels, bridging coagulation and inflammatory signaling pathways. Because of this dual role, they represent a particularly relevant target for RLN-2 during pregnancy and inflammatory states. Early observations suggested that RLN-2 may counteract hypercoagulability, raising the question of whether this peptide functions as an endogenous anticoagulant [44–46].

Experimental studies confirm that RLN-2 reduces platelet number and inhibits aggregation in a dose-dependent manner [44–46]. These changes are accompanied by striking alterations in megakaryocyte morphology, including cytoskeletal rearrangements that limit platelet release [44]. At the signaling level, RLN-2 suppresses the conformational changes and granular exocytosis required for platelet activation, effects mediated by the L-arginine–NO–cGMP pathway [44,45]. Importantly, platelets express RXFP1 as their only classic relaxin receptor, supporting the idea that RLN-2 directly modulates platelet function through this receptor [46]. Based on these findings, the ability of relaxin-2 to inhibit platelet aggregation was used as a functional assay to measure its bioactivity [46].

The role of GR in this context is less clear. Through GR expressed in megakaryocytes, glucocorticoids enhance platelet release, yet paradoxically diminish platelet aggregation [47,48]. Since platelets are anucleate, they cannot undergo classical genomic GR signaling; instead, potential effects of RLN-2–GR interaction could arise indirectly through megakaryocytes or via non-genomic pathways within platelets themselves. To date, however, no direct studies have addressed the contribution of RLN-2–GR signaling to platelet biology.

In summary, available evidence positions RLN-2 as a negative regulator of platelet production and activation, primarily via RXFP1 and NO–cGMP signaling [44–46]. This mechanism may contribute to its broader anti-thrombotic and immunomodulatory properties, with potential clinical implications in conditions characterized by hypercoagulability, such as pregnancy-related complications or thromboinflammatory disorders, including venous thromboembolism, atherosclerosis, and sepsis-associated coagulopathy.

## The influence of relaxin-2 on mastocytes

Mast cells, predominantly located in the connective tissue of the skin and mucosal membranes, are key effectors of allergic and inflammatory responses, releasing histamine and proteases that enhance immune activation and regulate inflammatory processes.

Evidence consistently shows that RLN-2 reduces mast cell degranulation and lowers circulating histamine levels [49,50]. Through RXFP1 activation, which is expressed in mast cells, RLN-2 suppresses the release of mediators such as chymase, tryptase, IL-6, and TNF-α [51]. These effects involve increased nitric oxide production, decreased intracellular Ca²^+^ concentration, and elevated cGMP, which together inhibit calcium-dependent exocytosis—a fundamental process that enables mast cells to rapidly and selectively release chemical mediators that modulate immune and inflammatory responses [49,50,52,53]. By limiting mediator release, RLN-2 exerts anti-inflammatory and immunosuppressive effects. Beyond these direct effects, RLN-2 reduces oxidative stress by stimulating endogenous NO production and limiting ROS accumulation, which may otherwise amplify mast cell activation and tissue damage. Improved coronary blood flow induced by RLN-2 may further facilitate ROS clearance, indirectly protecting against pro-inflammatory signaling [49]. In addition, RLN-2 activates the PI3K–Akt pathway, leading to inhibition of NF-κB and thereby reinforcing its anti-inflammatory profile [51].

An alternative explanation involves RLN-2 acting as a GR agonist. GR, expressed in mast cells, plays a key role in suppressing allergic and inflammatory responses, which underlies the clinical efficacy of corticosteroids in conditions such as asthma and atopic dermatitis—diseases strongly dependent on mast cell activation. Glucocorticoids are well known to stabilize mast cells and limit histamine release, potentially via rapid non-genomic GR pathways [54]. More specifically, this mechanism appears to operate similarly to RXFP1 by significantly limiting calcium influx, while also reducing the release of inflammatory cytokines and proteases. While this raises the possibility of dual signaling via RXFP1 and GR, direct mechanistic studies on RLN-2’s effects in this context are still lacking.

In summary, RLN-2 emerges as a potent inhibitor of mast cell activation, acting through RXFP1-dependent NO–cGMP and PI3K–Akt–NF-κB pathways, with additional protective effects linked to ROS neutralization [49–53]. A potential contribution of GR cannot be excluded, and elucidating this interaction may help refine future therapeutic applications of RLN-2 in allergic and inflammatory diseases.

## The impact of relaxin-2 on leukocytes

Leukocytes, or white blood cells, are a diverse group of immune cells responsible for defending the body against pathogens, removing damaged or malignant cells, and regulating inflammatory and immune responses. They are present both in the peripheral blood and in tissues, where some function as resident cells. Leukocytes are broadly classified into granulocytes (neutrophils, eosinophils, basophils), which mediate rapid innate responses; agranulocytes (lymphocytes, monocytes), which orchestrate adaptive immunity and differentiate into macrophages or dendritic cells; and antigen-presenting cells, which initiate and regulate immune responses. While GR is expressed in all leukocyte populations [55], RXFP1 has so far been reported in lymphocytes, monocytes, and macrophages [56], as well as in certain progenitor [56,57] and hematologic neoplasm (leukemia, lymphoma, and myeloma) cell lines [58].

Research indicates that relaxin-2 has a significant impact on white blood cells, which are crucial components of the immune system. Particularly, it may regulate the quantity of resident leukocytes, immune cells that are permanently present in tissues, such as those present in the endometrium. Notably, the action of relaxin-2 does not affect the number of CD3^+^ T lymphocytes, while simultaneously increasing the numbers of neutrophils, CD56^+^ natural killer (NK) cells, and CD68^+^ macrophages within the endometrium [59]. Interestingly, in the cardiac ischemia-reperfusion model, RLN-2 was shown to reduce leukocyte density and inhibit the expression of cytokines, such as IL-1β, IL-6, and monocyte chemoattractant protein-1 (MCP-1), without affecting TNFα expression [60].

Furthermore, it has been observed that relaxin-2 may exert indirect effects on immune cells by regulating the expression of certain important immunomodulatory proteins which protect the fetus from the maternal immune system during pregnancy; one such protein is glycodelin, a glycoprotein with immunosuppressive properties [61]. It has been found to suppress lymphocyte proliferation, the cytotoxic activity of NK cells, and Th1-type cytokine responses, as well as the induction of T-cell apoptosis and modulation of B cell function [62]. Evidence suggests that relaxin-2 is a potent stimulator of glycodelin synthesis both *in vitro* and *in vivo*, leading to an increase in glycodelin mRNA levels. Consequently, relaxin-2 activates the glycodelin transcriptome and enhances the concentration of glycodelin in plasma [63,64]. Although the specific mechanism underlying this phenomenon remains unknown, it has been noted that the glycodelin promoter contains glucocorticoid/progestagen response elements (GRE/PRE), whose activation is responsible for its increased expression [65,66]. Therefore, it is possible that relaxin-2 stimulates the production of glycodelin through the activation of GR, which subsequently binds to the GRE, triggering the transcription of the glycodelin gene. However, an increase in glycodelin expression was also obtained following administration of the GR antagonist mifepristone (RU486) [67]. Still, the properties of RU486 merit further study as it has been found to act as both an agonist and antagonist of GR [68].

In addition to its effects on glycodelin, relaxin-2 may modulate immune responses through galectins, a family of β-galactoside-binding proteins with diverse immunoregulatory functions. Galectin-1 and galectin-3, in particular, influence both innate and adaptive immunity by regulating T cell apoptosis, shaping cytokine profiles, and controlling the activation and migration of immune cells such as neutrophils and macrophages [69]. Upstream elements in the galectin-1 and galectin-3 genes include sequences responsive to glucocorticoids, suggesting a potential mechanism through which relaxin-2 could indirectly regulate galectin expression via GR signaling [70]. The relationship between relaxin-2 and galectins, especially galectin-3, has also been examined in cardiovascular contexts. In patients with atrial fibrillation, higher plasma levels of relaxin-2 were associated with increased Gal-3 concentrations in both left atrial and peripheral blood [70]. However, this correlation was not statistically significant after adjusting for confounding factors, including age, body mass index, and antihypertensive therapy, indicating that the link may be influenced by additional variables. While these observations suggest a potential interplay between relaxin-2 and galectin-3, further research is required to clarify the underlying mechanisms and their broader implications for immune regulation and disease pathogenesis.

### The impact of relaxin-2 on granulocytes

Granulocytes, a type of WBC with cytoplasmic granules, are key to innate and adaptive immunity. They contribute significantly to the process of inflammation and are necessary in the development of allergic reactions. The group comprises neutrophils, eosinophils, and basophils, all of which are influenced by relaxin-2 and play an important role in immune defense.

#### Neutrophils

Neutrophils, as the most abundant granulocytes, are frontline effectors of innate immunity and a major driver of inflammatory tissue damage. For this reason, their regulation by RLN-2 has attracted considerable attention.

Experimental studies demonstrate that RLN-2 reduces neutrophil activation by down-regulating CD11b expression and superoxide anion production in a dose-dependent manner, while simultaneously enhancing nitric oxide synthesis and lowering intracellular Ca²^+^ levels [71]. These effects impair degranulation, chemotaxis, and oxidative burst in response to classical activation stimuli such as fMLP and PMA. Importantly, chemically inactivated RLN-2 fails to reproduce these changes, and pharmacological inhibition of NO synthase reverses them, indicating a receptor-dependent mechanism involving RXFP1 and NO–cGMP signaling [71].

Beyond direct leukocyte regulation, RLN-2 also modulates endothelial–neutrophil interactions [72]. Treatment reduces the expression of P-selectin and VCAM-1 in activated endothelial cells, thereby attenuating neutrophil rolling, adhesion, and transendothelial migration. These effects, strictly dependent on RXFP1 and NO–cGMP signaling, collectively limit excessive neutrophil activation and tissue damage, providing anti-inflammatory and protective effects that are particularly relevant at the feto–maternal interface [72].

By inhibiting neutrophil activation, relaxin-2 may help regulate maternal immune responses, suggesting a potential role in early pregnancy processes relevant to preeclampsia, a disorder associated with excessive inflammation [71]. Additionally, lower placental RXFP1 expression has been observed in healthy individuals who later develop preeclampsia [73]. Relaxin-2 could support maternal immune tolerance and postpartum uterine recovery, and its dysfunction may increase antigen exposure and pregnancy complications.

The physiological significance of these mechanisms has been demonstrated *in vivo*. In models of myocardial ischemia–reperfusion, RLN-2 treatment lowers neutrophil infiltration, reduces myeloperoxidase (MPO) activity, decreases lipid peroxidation products such as malondialdehyde (MDA), and helps maintain calcium homeostasis [74]. MPO is an enzyme found in neutrophils that plays a key role in the immune response by producing reactive oxygen species, which are involved in the killing of pathogens [75]. Collectively, these actions limit oxidative stress and inflammatory damage while preserving myocardial integrity.

The phenotypic overlap between RLN-2 and glucocorticoid effects on neutrophils has prompted debate about underlying mechanisms. Both RLN-2 and glucocorticoids blunt neutrophil adhesion and integrin up-regulation following activation; dexamethasone, for example, reduces stimulus-induced CD11/CD18 up-regulation and neutrophil adhesion in several clinical and experimental settings [76,77]. Nevertheless, RLN-2’s dependence on NO signaling and the loss of effect with chemically inactivated peptide argue for a prominent RXFP1-mediated mechanism in neutrophils.

Thus, current evidence indicates that RLN-2 suppresses neutrophil activation and recruitment during pregnancy and inflammation through RXFP1-dependent, NO–cGMP-mediated pathways, while a contribution of GR remains possible but unconfirmed [71–74].

#### Basophils

Basophils, though the least abundant granulocytes, play an outsized role in allergic inflammation through rapid release of histamine, leukotrienes, and cytokines. Despite their importance, data on RLN-2 modulation of basophils are relatively scarce.

RLN-2 treatment reduces the expression of CD63, a major marker of basophil activation, in a dose-dependent manner and prevents anaphylactic degranulation in response to both immunogenic stimuli (anti-IgE antibodies) and non-immunogenic stimuli, such as phorbol 12-myristate 13-acetate (PMA) [78]. These findings indicate that RLN-2 may have therapeutic potential in dampening allergic and anaphylactic responses by limiting basophil activation.

Mechanistically, RLN-2 attenuates the rise in intracellular calcium required for granule release during basophil activation [78]. Electron microscopy studies further demonstrate that RLN-2 maintains basophils in a quiescent state, preserving granule integrity and preventing morphological hallmarks of degranulation. Importantly, RLN-2 inhibits both immunologic activation via IgE-bound FcεRI receptors and non-immunologic activation mediated by PKC signaling, indicating a broad regulatory effect on basophil function [78,79].

The inhibitory actions of RLN-2 are closely linked to its enhancement of calcium/calmodulin-dependent nitric oxide synthase activity in basophils, leading to increased endogenous NO production [78]. The centrality of NO is supported by the finding that NOS inhibitors (e.g., L-NMMA) or guanylate cyclase inhibitors (e.g., ODQ) markedly reduce the effects of RLN-2, while the NO donor sodium nitroprusside (SNP) can replicate them. Chemically inactivated RLN-2 fails to inhibit basophil activation, further implicating RXFP1-mediated signaling [78].

Interestingly, glucocorticoids inhibit the activation of peripheral blood basophils by a non-genomic pathway, which also prevents the up-regulation of CD63 expression on the basophil surface [80]. Glucocorticoids effectively reduce the release of inflammatory mediators from basophils, thereby modulating their activity and the associated inflammatory response [80]. These findings suggest that relaxin-2, acting via RXFP1 and possibly, but less likely, involving GR-dependent mechanisms, can effectively restrain basophil activation by regulating calcium signaling and boosting nitric oxide production, pointing to its potential role in controlling allergic and inflammatory responses [78].

#### Eosinophils

Eosinophils contribute to host defense against parasites but are best known for their pathogenic role in allergic disorders and asthma. Their regulation by RLN-2 has not been extensively studied, yet available reports provide intriguing clues. One study examined whether relaxin-2 influences collagen remodeling in the cervical tissue of rats, at least in part by promoting eosinophilic invasion and degranulation; however, no significant changes in eosinophil morphology or function were observed following treatment [81]. This may be in line with findings from other studies suggesting that relaxin-2 may actually reduce degranulation of other granulocytes [78]. It has also been proposed that relaxin-2 may exert anti-fibrotic effects by modulating the secretion of IL-4 and IL-5 by Th-2 cells and the expression of IL-13 by eosinophils [82]. There is hence a need for further research to better understand the role of relaxin-2 in eosinophils and their functions.

### The effect of relaxin-2 on agranulocytes

Relaxin-2 has been shown to exert significant effects on peripheral blood mononuclear cells (PBMCs)—agranulocytes lacking visible cytoplasmic granules. This group includes lymphocytes (T, B, and NK cells), which mediate adaptive immune responses, and monocytes, which differentiate into macrophages and dendritic cells in tissues. Together, agranulocytes play central roles in pathogen elimination, phagocytosis, and regulation of inflammatory and immune responses.

PBMCs express RXFP1 at the mRNA level, and RLN-2 treatment increases the secretion of pro-inflammatory cytokines, such as TNF-α and interleukin-1β (IL-1β) [83]. RLN-2 also induces a concentration-dependent accumulation of cAMP in PBMCs, affecting key biological functions including adhesion and migration [84]. Collectively, these observations identify RLN-2 as a novel stimulator of leukocyte recruitment, suggesting a potential role in promoting the accumulation and retention of immune cells at sites of inflammation or tissue repair.

In addition to modulating PBMC behavior, RLN-2 influences the differentiation and function of CD4^+^ T cells. It promotes the polarization of CD4^+^ T cells toward effector T helper 1 (Th1) cells, enhancing their production of interferon-gamma (IFN-γ), a key cytokine for activating macrophages, mediating antibody-dependent cellular cytotoxicity, and facilitating delayed-type hypersensitivity responses, which appears a few days after exposure to the antigen [85,86]. In T-cells, RLN-2 does not affect interleukin-4 (IL-4) production, indicating limited impact on Th2-mediated humoral responses. In CD4^+^ T cell clones, RLN-2 increases both IFN-γ mRNA expression and IFN-γ secretion following T-cell receptor stimulation, suggesting a direct effect on Th1 differentiation and function, potentially independent of IL-12 or IFN-α release by antigen-presenting cells [85,86]. However, little is known of any such relationship between the effect of relaxin-2 on its cognate receptor. Interestingly, in this context, activation of the GR by glucocorticoids tends to redirect Th differentiation toward Th2 rather than Th1 [87].

In systemic lupus erythematosus (SLE), agranulocytes, particularly lymphocytes and monocytes, are central to disease pathology through excessive activation and autoantibody production, which drive tissue damage and immune dysregulation. Within this context, RLN-2 shows modulatory effects on T cells and monocytes but appears to exert minimal influence on other immune cell populations. Serelaxin, a recombinant form of RLN-2, does not alter B lymphocyte counts or plasma levels of anti-dsDNA autoantibodies, indicating a limited impact on B cells and cytotoxic T lymphocytes in this autoimmune setting [88].

#### Expression of relaxin family peptide receptors among MPS cells

Within the mononuclear phagocyte system (MPS), which includes monocytes, macrophages, and dendritic cells, the effects of RLN-2 have been studied most extensively. Monocytes are circulating leukocytes that differentiate into macrophages or dendritic cells after migrating into tissues. Macrophages eliminate pathogens by phagocytosis and present antigens to T cells, while dendritic cells serve as specialized antigen-presenting cells initiating adaptive responses. In this context, the influence of RLN-2 has been characterized most thoroughly in the THP-1 monocyte/macrophage cell line.

The expression of relaxin family peptide receptors varies depending on both cell type and differentiation state. In THP-1 cells, RXFP1 is highly expressed, whereas RXFP3 and RXFP4 are present at lower levels [46,89,90]. Reports regarding RXFP2 expression in THP-1 cells are inconsistent [46,90]. *In vivo*, CD68^+^ macrophages in a mouse model of myocardial infarction also express RXFP1 [91]. In human decidual macrophages (DMs) obtained from patient samples, RXFP1 is expressed and localizes both to the cell surface and intracellular compartments, although its levels are lower than in THP-1 cells [40]. These DMs play a central role in immune regulation, significantly influencing embryo implantation, as well as the maintenance and progression of pregnancy. Notably, differentiated THP-1 cells exhibit the lowest RXFP1 expression [40], while macrophages within the tumor microenvironment show the highest RXFP1 levels [92].

#### Differential effects of relaxin-1 and relaxin-2 on immune cell infiltration

Immune cell infiltration into tissues is a critical step shaping both inflammation and tissue repair. In this context, relaxins emerge as important regulators of leukocyte trafficking [84]. For RLN-2, several studies point to a consistent ability to limit macrophage accumulation [91]. In myocardial tissue, RLN-2 reduced macrophage infiltration without altering neutrophil dynamics [91], while in a liver transplantation model, it decreased both macrophage and neutrophil entry [93]. These effects parallel observations for glucocorticoids, which also diminish macrophage infiltration in inflamed tissues [94]. Interestingly, RLN-1 appears to act differently. In tumor models, gene delivery of RLN-1 promoted the infiltration of cytotoxic and antigen-presenting immune cells (CD8^+^ T cells, dendritic cells, NK cells) and simultaneously reduced Tregs, effectively shifting the tumor microenvironment toward an anti-tumor, immunostimulatory phenotype [8,92].

Taken together, these findings highlight a dual picture: RLN-2 primarily acts to restrain excessive infiltration, thereby reducing tissue damage, whereas RLN-1 can amplify the presence of effector immune cells in certain contexts. The balance between these effects may depend on context-specific regulation of immune responses, reflecting the distinct physiological roles attributed to RLN-1 and RLN-2.

#### Relaxin-2 as a modulator of tumor microenvironment dynamics

The tumor microenvironment (TME) represents a complex immunological niche where infiltrating leukocytes can either restrain or promote tumor growth. Within this landscape, RLN-2 emerges as a key modulator of macrophage function.

Several studies indicate that RLN-2 promotes the differentiation of infiltrating monocytes into tumor-associated macrophages (TAMs), cells known to support tumor progression by stimulating angiogenesis, immune evasion, and metastasis [95]. In both a murine HER2^+^ breast cancer model and an *ex vivo* brain metastasis system, RLN-2 increased TAM infiltration via RXFP1 activation, thereby fostering conditions that favor tumor growth and colonization [96]. Importantly, this effect appears to rely on cAMP-dependent pathways and nitric oxide signaling, which together help anchor macrophages within the TME [95,96].

These findings stand in contrast to the more anti-inflammatory, tissue-protective actions of RLN-2 observed in non-malignant contexts. In tumors, the same signaling axes that suppress inflammation may instead reinforce an immunosuppressive environment.

#### Relaxin-2–mediated adhesion, migration, and cytokine production

The ability of monocytes and macrophages to adhere, migrate, and secrete cytokines is central to their role in immunity and tissue homeostasis. RLN-2 has been shown to modulate each of these processes, often through RXFP1-mediated cAMP signaling but with important overlaps with GR pathways.

RLN-2 enhances leukocyte adhesion and migration. It promotes both intercellular adhesion, facilitating interactions with other immune cells, and adhesion to extracellular matrix components, which supports tissue retention and movement. In THP-1 cells, incubation with RLN-2 increased adhesion to tissue culture plastic by 1.5-fold and nearly doubled migration [84]. These migratory effects were associated with chemotaxis toward MCP-1, a chemokine critical for recruiting leukocytes to sites of inflammation or cancer, and were shown to depend on the RXFP1/Gαs/adenylate cyclase/cAMP signaling axis [84,89].

Detailed kinetic studies show that RLN-2 elicits a biphasic cAMP response: an initial surge within 1–2 minutes of stimulation, followed by a second, delayed peak around 20 min [90]. The early rise reflects classical GPCR signaling, whereas the later peak requires partial PI3K activity—even though PI3K is not typically coupled with G-protein activation in this setting. In THP-1 cells, RLN-2 also triggers a transient MAPK activation that peaks at 10 min and returns to baseline by 30 minutes, a process that may contribute to the two-phase cAMP pattern [97,98]. Notably, pharmacologic PI3K inhibition suppresses both cAMP peaks—by roughly 30% for the first and 70% for the second—underscoring an auxiliary role for PI3K [90]. However, this effect is unlikely to involve GR signaling, as GR activity typically inhibits PI3K [99]. Supporting the idea of cell-type–specific pathways, Anand-Ivell and colleagues reported that in THP-1 cells, RXFP1-mediated cAMP production occurs independently of MAPK and PI3K activation, but that PKA and, subsequently, MAPK may act as downstream effectors of the RXFP1–cAMP pathway [89]. Likewise, in HEK293T-RXFP1 cells, RLN-2–induced signaling occurred without detectable tyrosine kinase activity, further emphasizing the diversity of RLN-2 signaling [89].

RLN-2 reduces pro-inflammatory cytokine output in MPS cells. In THP-1 cells, RLN-2 decreased IL-6 and IL-1β mRNA, while TNF-α and IL-10 remained unaffected [91]. In decidual macrophages, RLN-2 reduced CSF2 and IL-8 secretion, particularly in the absence of immunogenic stimuli, thereby limiting neutrophil chemotaxis [40]. In LPS-stimulated macrophages, RLN-2 suppressed acute-phase cytokines (IL-1, IL-6, TNF-α) by up to 40% [33,37]. At the same time, RLN-2 selectively spared certain adhesion molecules: while it reduced MCP-1 expression and dampened THP-1 adhesion to endothelial cells, it did not affect VLA-4 or PECAM expression, suggesting targeted rather than global suppression of leukocyte recruitment [38]. Interestingly, relaxin-1 gene therapy also down-regulated expression of Th2 cytokines (IL4, IL6, and IL10) and chemokines (CCL2 and CCL5), while increasing Th1 cytokines (IL-12 and IFN-γ) in the liver metastatic lesion *in vivo* [8]. RLN-1 gene therapy can reprogram the liver metastatic niche from an immunosuppressive environment to one that stimulates immune activity.

In wound-resident macrophages, RLN-2 induced VEGF and bFGF expression, supporting angiogenesis and tissue repair [100]. In contrast, glucocorticoids suppress VEGF in monocytes/macrophages [101], marking an important divergence between RLN-2 and GR signaling. RLN-2 also up-regulated MMP-9, but not MMP-2, through NF-κB activation, linking it to tissue remodeling and cancer progression [102].

Serelaxin increased Notch-1 intracellular domain (NICD) and Hes1 expression, while reducing pro-inflammatory mediators (pIkBa, MCP-1, IL-1β, CXCL1, CXCL2) and promoting Arg1 and IL-1 [93]. Arg1 is an anti-inflammatory enzyme and a marker of M2 macrophages that suppresses immune responses, whereas IL-1 is a potent pro-inflammatory cytokine that activates and regulates immune activity. These effects mirror GR-dependent immunoregulation, where GR activation also induces Arg1 and IL-10 while suppressing the same pro-inflammatory mediators [103–106]. Many of RLN-2’s effects resemble glucocorticoid action: suppression of IL-1β, IL-6, and MCP-1, induction of Arg1 and IL-10, and down-regulation of chemokines that recruit neutrophils [40,93,103–106]. Yet clear differences exist: unlike glucocorticoids, RLN-2 enhances VEGF and MMP-9, suggesting that its tissue effects extend beyond classic GR biology.

Altogether, RLN-2 orchestrates a hybrid signaling program in MPS cells. RXFP1/cAMP drives migration and chemotaxis, while GR-like mechanisms dampen inflammatory cytokine production. The divergence in pro-angiogenic outputs (VEGF, MMP-9) indicates that RLN-2 cannot be equated simply with glucocorticoids. There is the need for further mechanistic studies that disentangle the respective contributions of RXFP1 and GR.

#### The role of relaxins in macrophage polarization

Macrophages are highly plastic immune cells capable of adopting a wide spectrum of activation states in response to microenvironmental signals. The widely used classification into M1-like (classically activated) and M2-like (alternatively activated) phenotypes largely originates from *in vitro* studies, where defined stimuli such as IFN-γ and LPS drive an M1-like, pro-inflammatory profile, while IL-4 and IL-13 promote an M2-like, anti-inflammatory and tissue-repairing state [107]. M0 macrophages are precursors of M1-like and M2-like macrophages. Within this context, relaxin-2 plays a crucial role in modulating macrophage polarization and inflammatory responses.

RLN-2 has been shown to significantly reduce cell infiltration while increasing the expression of M2-like macrophage markers associated with anti-inflammatory responses, such as MRC, IL-4, arginase, IL-10, Ym1, and CX3CR1 [108]. Conversely, RLN-2 treatment decreased the expression of pro-inflammatory M1-like macrophage markers, including iNOS, TNF-α, CCL-3, and IL-23. *In vitro*, incubation with RLN-2 reduced iNOS and increased arginase expression across all three macrophage populations (M0, M1, M2), suggesting that RLN-2 facilitates a transition from M1-like to M2-like states. *In vivo* studies further confirmed that RLN-2 promotes the polarization of both M0 and M1-like macrophages toward the M2 phenotype [108]. Conversely, relaxin-1 gene delivery raised the M1/M2 macrophage ratio within liver metastasis, indicating its pro-inflammatory properties [8].

Glucocorticoids exhibit partly overlapping effects. Upon wounding, they inhibit differentiation toward the pro-inflammatory M1 phenotype without impeding macrophage migration, thereby supporting tissue repair and resolution of inflammation [109]. Glucocorticoids also interact with IL-4 to enhance Ym1 expression in alternatively activated myeloid cells, reinforcing M2 polarization [110]. In addition, they suppress transcription of the pro-inflammatory gene CCL3 (MIP-1α) while enhancing *MRC1* (also known as CD206) expression in macrophages, further promoting a tissue-repair phenotype [111,112].

Mechanistic studies indicate that RLN-2 reduces the expression of several key molecules involved in inflammatory signaling [108]. It down-regulates TLR4, a key receptor that senses bacterial components and triggers pro-inflammatory cytokine production and immune cell activation and its adaptor MyD88. Thereby, it attenuates NF-κB activation, as reflected by reduced NF-κB (p65) and phospho-NF-κB (p-p65) at both mRNA and protein levels. These effects were observed across M0, M1, and M2 macrophages in the unilateral ureteral obstruction (UUO) model. Notably, RLN-2–induced M2 polarization is blocked by the TLR4 antagonist TAK-242, confirming that inhibition of TLR4 signaling is central to this process [108].

The interplay between RLN-2 and GR signaling adds another layer of complexity. Western blot analyses revealed that differentiated THP-1 cells express higher GR and lower RXFP1 levels compared with undifferentiated THP-1 cells and differentiated macrophages, suggesting that RLN-2 may primarily act via RXFP1 in undifferentiated cells, while GR contributes in differentiated states [33]. In decidual macrophages, the reduction in CSF2 and IL-8 secretion after RLN-2 treatment was abolished by RU486, supporting the notion that RLN-2 can function as a GR agonist [40].

Further evidence of convergence between RLN-2 and GR signaling includes the ability of RLN-2 to activate CREB, a transcription factor known to enhance GR signaling [98]. The RLN-2–induced rise in intracellular cAMP may further augment GR expression via CREB activation. Conversely, GR typically inhibits PI3K activity, acting as a brake on this pathway [113]. RLN-2 was also found to suppress endotoxin-induced AP-1 activation, pointing to broad regulation of inflammatory transcriptional programs [37]. Interestingly, in bone marrow–derived macrophages (BMDMs), serelaxin did not increase RXFP1 or GR expression, leaving the precise mechanisms unresolved [93].

Taken together, these findings demonstrate that RLN-2 promotes macrophage polarization toward an M2-like phenotype by inhibiting TLR4–NF-κB signaling and enhancing CREB-dependent transcription, while partially overlapping with GR-mediated regulation.

#### The impact of relaxin-1 on antigen-presenting cells

Antigen-presenting cells (APCs) are key to the immune system, processing and presenting antigens to T cells. This group includes dendritic cells (DCs), which connect the innate and adaptive immune systems. DCs capture antigens via endocytosis and present peptide fragments on MHC molecules to T cells, activating them to fight infections and tumors.

**Figure 3:**
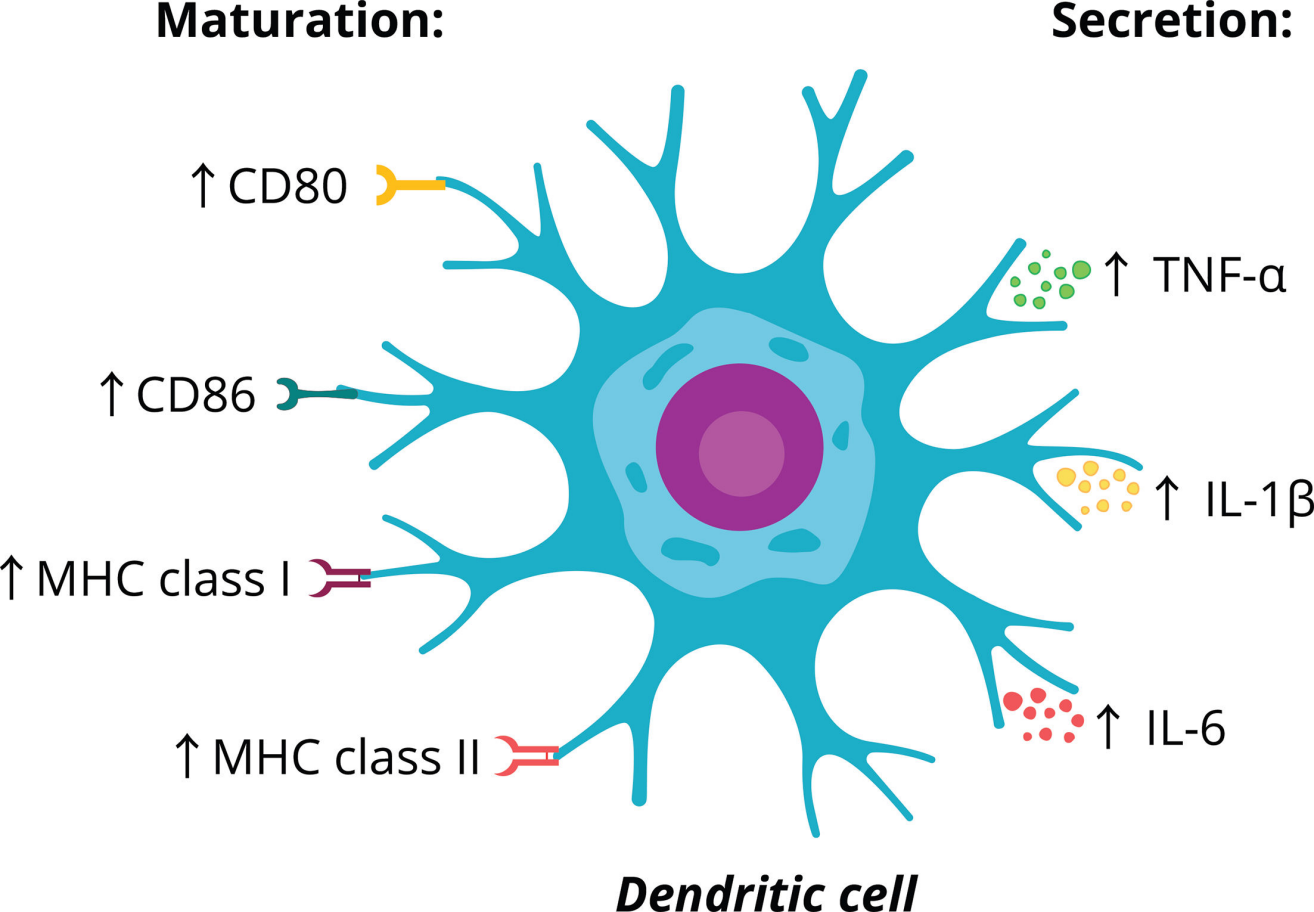
The impact of relaxin-1 on dendritic cells. ↑ up-regulation. CD80, cluster of differentiation 80; CD86, cluster of differentiation 86; IL-1β, interleukin 1β; IL-6, interleukin 6; MHC, major histocompatibility complex; TNF-α, tumor necrosis factor α.

Relaxin-1, like relaxin-2, significantly affects the MPS by inducing maturation in APCs, particularly dendritic cells. It promotes the maturation of immature DCs, increasing surface markers like CD80, CD86, and MHC class I and II molecules essential for T cell activation [114]. Mature DCs activate helper and cytotoxic T cells by presenting antigens, enhancing the immune response. This is critical in settings where immune responses are weak or need to be artificially enhanced, such as in cancer or chronic infections. Mature DCs are essential for initiating cytotoxic CD8 + T cell responses and CD4 + helper T cell responses, both of which are key to clearing tumors and infected cells. Relaxin-1 also increases the secretion of pro-inflammatory cytokines (TNF-α, IL-1β, and IL-6) from treated DCs, indicating it improves both the morphology and function of APCs (Figure 3). This probably occurs through its specific receptor, RXFP1, expressed on DCs [114].

## Summary and future prospects

The relaxin family of peptide hormones has long been studied in the context of pregnancy. However, over the past few decades, due to their multifaceted action, research has expanded to explore their functions in other medical areas such as oncology, cardiology, and rheumatology [12]. It has become evident that relaxin peptides also have a significant impact on blood cells.

RLN-2 exerts inhibitory effects on various blood cell types. In megakaryocytes and platelets, it lowers cell counts and reduces aggregation; in mast cells and basophils, it prevents degranulation; and in neutrophils, it limits activation [44,45,49,71,78]. At the same time, RLN-2 stimulates glycodelin synthesis and promotes CD4^+^ T cell differentiation into Th1 effectors, thereby enhancing IFN-γ production [64,85]. Within the myeloid lineage, RLN-2 modulates macrophage infiltration and shapes the tumor microenvironment by increasing tumor-associated macrophages [91,95]. It also suppresses pro-inflammatory cytokines and drives polarization toward anti-inflammatory, M2-like phenotypes [37,40,84,91,108]. By contrast, RLN-1 appears to enhance immune activity, for example, by inducing DC maturation [114].

Relaxin signaling, primarily through RXFP1, has been implicated in disease contexts such as leukemia and preeclampsia, with elevated receptor expression linked to poorer survival in leukemia patients and decreased placental expression associated with later development of preeclampsia [32,71,73]. These observations underscore the importance of relaxin pathways in both health and disease.

An emerging theme is the dual receptor engagement of RLN-2. While RXFP1 explains many of its canonical effects—on cAMP signaling, cell migration, and angiogenesis—evidence also suggests RLN-2 can act as a GR agonist. In some models, RLN-2 increased GR expression, suppressed pro-inflammatory cytokines, and induced M2 polarization in ways that overlap with glucocorticoid biology [40,108]. Yet, differences are equally striking: RLN-2 enhances VEGF and MMP-9 expression, activities not shared with glucocorticoids [100–102,109]. The most significant similarities and differences between relaxin-2 and glucocorticoids are summarized in Table 1.

**Table 1:** Similarities and differences in relaxin-2 and glucocorticoids action on blood cellular components

	Relaxin-2	Glucocorticoids
Platelets	↓ Number of circulating platelets [44]	↑ Number of young platelets [47]
	↓ Platelet aggregation [46]	↓ Platelet aggregation [48]
Megakaryocytes	↓ Production and release of platelets [44,45]	↑ Production and release of platelets [47]
	↓ Exocytosis [45]	↑ Exocytosis [47]
	Rearrangement of the actin microfilament [44]	Cytoskeleton remodeling [47]
Mast cells	↓ Degranulation by increase in intracellular Ca^2+^ [49–52]	↓ Histamine exocytosis by raise of intracellular Ca^2+^ [54]
Erythrocytes	↓ Circulating count [8]	↑ Circulating count [115]
Basophils	↓ Basophil activation (CD63 expression) [78]	↓ Non-genomic basophil activation (CD63 expression) [80]
Neutrophils	↓ Activation (CD11b expression) [71]	↓ Activation (CD11b expression) [77]
	↓ Adhesion [72,74]	↓ Adhesion [77]
	↓ Recruitment [74]	↓ Recruitment [116]
Macrophages	↓ Infiltration [91]	↓ Infiltration [94]
	↑ Differentiation toward M2 [108]	↑ Differentiation toward M2 [109]
	↓ IL-1 β, IL-6, IL-8, IL-23, MCP-1, pIkBa, TNF-α [40,60,93,108]	↓ IL-1β, IL-6, IL-8, IL-23, MCP-1, pIkBa, TNF-α [103,104,117–120]
	↓ CSF2, CXCL1, CXCL2, CCL-3 [40,93,108]	↓ CSF2, CXCL1, CXCL2 (*in airway smooth muscle cells and fibroblasts*), CCL-3 [106,111]
	↑ Arg1, IL-4, IL-10, Ym1 [93,108]	↑ Arg-1, IL-4, IL-10, Ym1 [105,110]
	↑ MRC (CD206) [108]	↑ MRC (CD206) [112]
	↑ VEGR [100]	↓ VEGF [101]
	↑ bFGF [100]	↓ bFGF (*colon carcinoma cells*) [121]
	↑ CX3CR1 [108]	No data
	↓ Adhesion [38]	↓ Adhesion [109]
	↓ Recruitment (MCP-1) [38]	↓ Recruitment (MCP-1) [103]
	↑ MMP-9 [102]	↓ MMP-9 [109]
T cells	↑ Differentiation (Th1) [85,86]	↑ Differentiation (Th2) [87]
	↑ Secretion (IFN-γ) [85,86]	↓ Cytokine production [117]
B cells	~ Number in the blood [88]	↓ Number in the blood [117]

↓ down-regulation, ↑ up-regulation, ~ no change

Molecular studies add further nuance. RLN-2 induces phosphorylation of Ser211 in GR, similar to glucocorticoids, but glucocorticoid signaling also involves additional phosphorylation sites (Ser203, Ser226, Ser404) [37,122,123]. Thus, while RLN-2 may activate GR, it likely does so in a manner distinct from classical ligands, leading to partially overlapping but non-identical outcomes. Whether RLN-2 consistently binds GR with high affinity and drives transcription of GRE-regulated genes remains a matter of debate, with some studies reporting robust nuclear translocation and others finding negligible or context-dependent activity [11,38,40].

Beyond its biological effects, the systemic safety profile of RLN-2 is an important consideration. Clinical studies so far indicate that serelaxin is well tolerated, with intravenous administration showing a favorable safety profile in acute [124,125] and chronic heart failure [126], cirrhosis [127], and post-date pregnancy [128], as well as subcutaneous application in diffuse scleroderma [129]. Nevertheless, immunogenicity remains a concern: healthy individuals possess pre-existing RLN-2–reactive CD4^+^ T cells (0.61 per million), raising the possibility that repeated dosing could elicit anti-RLN-2 antibodies [130]. Alternative approaches, such as gene delivery, have demonstrated minimal toxicity even in combination with immune checkpoint blockade [8,92], and may represent a promising way to circumvent immunogenicity associated with repeated injections. Certain pharmacological properties of relaxin-2 should also be considered—notably its antithrombotic activity, which may impose restrictions on its clinical dose [44,45].

We acknowledge that most of the evidence presented in this manuscript derives from *in vitro* and animal studies, and it is well recognized that immune responses in humans may differ substantially from those observed in such models. This underscores the need for clinical studies to investigate the immunomodulatory properties of relaxin-1 and relaxin-2. Although growing evidence suggests that RLN-2 may function as a GR agonist, the extent to which these effects occur independently of RXFP1 remains controversial. Importantly, RLN-2 appears to play a dual role depending on tissue context: in healthy tissues, it can exert protective and pro-regenerative effects, whereas in certain tumor microenvironments, its immunosuppressive actions might inadvertently favor tumor growth. Nevertheless, this complexity does not diminish the strong translational potential of RLN-2 itself. Its pleiotropic properties provide a clear rationale for testing RLN-2 in clinical contexts where excessive inflammation, vascular dysfunction, and immune dysregulation drive morbidity. Beyond its previous evaluation in acute heart failure, serelaxin or next-generation RLN-2 analogs could be explored in autoimmune diseases such as rheumatoid arthritis and systemic lupus erythematosus, in fibrotic conditions including systemic sclerosis and chronic kidney disease, and in pregnancy-related complications such as preeclampsia. Early clinical trials of serelaxin have provided preliminary evidence of safety; future early-phase trials can focus on immunogenicity and biomarker-based assessments to define its therapeutic potential.

## Open Access

This article has been published open access under our Subscribe to Open programme, made possible through the support of our subscribing institutions, learn more here: https://portlandpress.com/pages/open_access_options_and_prices#conditional

## Abbreviations

DEX: dexamethasone
GR: glucocorticoid receptor
GRE: glucocorticoid response element
RLN-1: relaxin-1
RLN-2: relaxin-2
RXFP1: relaxin family peptide receptor 1

## References

[1] Goldsmith L.T. Weiss G. Steinetz B.G 1995 Relaxin and its role in pregnancy Endocrinol. Metab. Clin. North Am. 24 171 186 10.1016/S0889-8529(18)30058-6 7781625

[2] Hisaw F.L 1926 Experimental relaxation of the pubic ligament of the guinea pig Exp. Biol. Med. (Maywood) 23 661 663 10.3181/00379727-23-3107

[3] MacLennan A.H 1983 The role of relaxin in human reproduction Clin. Reprod. Fertil. 2 77 95 6322955

[4] Zhang D. Wang Y. Yu S. Niu H. Gong X. Miao X 2015 Serum relaxin levels as a novel biomarker for detection of acute myocardial infarction Int. J. Clin. Exp. Med. 16937 16940 26629247 PMC4659135

[5] Stewart D.R 2019 Commercial immunoassays for human relaxin-2 Mol. Cell. Endocrinol. 487 94 97 10.1016/j.mce.2019.01.004 30633956

[6] Rais Y. Drabovich A.P 2024 Identification and Quantification of Human Relaxin Proteins by Immunoaffinity-Mass Spectrometry J. Proteome Res. 23 2013 2027 10.1021/acs.jproteome.4c00027 38739617

[7] Sherwood O.D 2004 Relaxin’s physiological roles and other diverse actions Endocr. Rev. 25 205 234 10.1210/er.2003-0013 15082520

[8] Hu M. Wang Y. Xu L. An S. Tang Y. Zhou X. et al 2019 Relaxin gene delivery mitigates liver metastasis and synergizes with check point therapy Nat. Commun. 10 2993 10.1038/s41467-019-10893-8 31278269 PMC6611764

[9] Bathgate R.A. Ivell R. Sanborn B.M. Sherwood O.D. Summers R.J 2006 International Union of Pharmacology LVII: recommendations for the nomenclature of receptors for relaxin family peptides Pharmacol. Rev. 58 7 31 10.1124/pr.58.1.9 16507880

[10] National Center for Biotechnology Information 2024 PubChem Compound Summary for CID 71300770, Human relaxin PubChem

[11] Bathgate R.A.D. Hsueh A.J.W. Sherwood O.D 2006 Physiology and Molecular Biology of the Relaxin Peptide Family In editorKnobil and Neill’s Physiology of Reproduction ( Neill J.D. ed 3rd edition ed pp 679 768 St Louis: Academic Press 10.1016/B978-012515400-0/50021-X

[12] Samuel C.S. Royce S.G. Hewitson T.D. Denton K.M. Cooney T.E. Bennett R.G 2017 Anti-fibrotic actions of relaxin Br. J. Pharmacol. 174 962 976 10.1111/bph.13529 27250825 PMC5406285

[13] Liu C. Eriste E. Sutton S. Chen J. Roland B. Kuei C. et al 2003a Identification of Relaxin-3/INSL7 as an endogenous ligand for the orphan g-protein-coupled receptor GPCR135 Journal of Biological Chemistry 278 50754 50764 10.1074/jbc.M308995200 14522968

[14] Liu C. Chen J. Sutton S. Roland B. Kuei C. Farmer N. et al 2003 Identification of relaxin-3/INSL7 as a ligand for GPCR142 J. Biol. Chem. 278 50765 50770 10.1074/jbc.M308996200 14522967

[15] Hsu S.Y. Nakabayashi K. Nishi S. Kumagai J. Kudo M. Sherwood O.D et al 2002 Activation of orphan receptors by the hormone relaxin Science 295 671 674 10.1126/science.1065654 11809971

[16] Zhang S.-S. Larrabee L. Chang A.H. Desai S. Sloan L. Wang X. et al 2024 Discovery of RXFP2 genetic association in resistant hypertensive men and RXFP2 antagonists for the treatment of resistant hypertension Sci. Rep. 14 13209 10.1038/s41598-024-62804-7 38851835 PMC11162469

[17] Dschietzig T. Bartsch C. Richter C. Laule M. Baumann G. Stangl K 2003 Relaxin, a pregnancy hormone, is a functional endothelin-1 antagonist: attenuation of endothelin-1-mediated vasoconstriction by stimulation of endothelin type-B receptor expression via ERK-1/2 and nuclear factor-kappaB Circ. Res. 92 32 40 10.1161/01.res.0000051884.27117.7e 12522118

[18] Dschietzig T. Bartsch C. Stangl V. Baumann G. Stangl K 2004 Identification of the pregnancy hormone relaxin as glucocorticoid receptor agonist FASEB J. 18 1536 1538 10.1096/fj.03-1120fje 15289446

[19] Lockett J. Inder W.J. Clifton V.L 2024 The glucocorticoid receptor: isoforms, functions, and contribution to glucocorticoid sensitivity Endocr. Rev. 45 593 624 10.1210/endrev/bnae008 38551091 PMC11244253

[20] Quatrini L. Ugolini S 2021 New insights into the cell- and tissue-specificity of glucocorticoid actions Cell. Mol. Immunol. 18 269 278 10.1038/s41423-020-00526-2 32868909 PMC7456664

[21] Halls M.L. Bathgate R.A.D. Sutton S.W. Dschietzig T.B. Summers R.J 2015 International Union of Basic and Clinical Pharmacology. XCV. Recent advances in the understanding of the pharmacology and biological roles of relaxin family peptide receptors 1-4, the receptors for relaxin family peptides Pharmacol. Rev. 67 389 440 10.1124/pr.114.009472 25761609 PMC4394689

[22] Erlandson S.C. Rawson S. Osei-Owusu J. Brock K.P. Liu X. Paulo J.A. et al 2023 The relaxin receptor RXFP1 signals through a mechanism of autoinhibition Nat. Chem. Biol. 19 1013 1021 10.1038/s41589-023-01321-6 37081311 PMC10530065

[23] Leo C.H. Jelinic M. Ng H.H. Marshall S.A. Novak J. Tare M. et al 2017 Vascular actions of relaxin: nitric oxide and beyond Br. J. Pharmacol. 174 1002 1014 10.1111/bph.13614 27590257 PMC5406296

[24] Chow B.S.M. Chew E.G.Y. Zhao C. Bathgate R.A.D. Hewitson T.D. Samuel C.S 2012 Relaxin signals through a RXFP1-pERK-nNOS-NO-cGMP-dependent pathway to up-regulate matrix metalloproteinases: the additional involvement of iNOS PLoS ONE 7 e42714 10.1371/journal.pone.0042714 22936987 PMC3425563

[25] Tapia Cáceres F. Gaspari T.A. Hossain M.A. Samuel C.S 2022 Relaxin Inhibits the Cardiac Myofibroblast NLRP3 Inflammasome as Part of Its Anti-Fibrotic Actions via the Angiotensin Type 2 and ATP (P2X7) Receptors Int. J. Mol. Sci. 23 7074 10.3390/ijms23137074 35806076 PMC9266307

[26] Mookerjee I. Hewitson T.D. Halls M.L. Summers R.J. Mathai M.L. Bathgate R.A.D. et al 2009 Relaxin inhibits renal myofibroblast differentiation via RXFP1, the nitric oxide pathway, and Smad2 FASEB J. 23 1219 1229 10.1096/fj.08-120857 19073841

[27] Bennett R.G. Heimann D.G. Singh S. Simpson R.L. Tuma D.J 2014 Relaxin decreases the severity of established hepatic fibrosis in mice Liver Int. 34 416 426 10.1111/liv.12247 23870027 PMC3843971

[28] Singh S. Simpson R.L. Bennett R.G 2015 Relaxin Activates Peroxisome Proliferator-activated Receptor γ (PPARγ) through a Pathway Involving PPARγ Coactivator 1α (PGC1α) Journal of Biological Chemistry 290 950 959 10.1074/jbc.M114.589325 25389293 PMC4294522

[29] Ahmad N. Wang W. Nair R. Kapila S 2012 Relaxin induces matrix-metalloproteinases-9 and -13 via RXFP1: induction of MMP-9 involves the PI3K, ERK, Akt and PKC-ζ pathways Mol. Cell. Endocrinol 363 46 61 10.1016/j.mce.2012.07.006 22835547 PMC3447121

[30] Heng K. Ivell R. Wagaarachchi P. Anand-Ivell R 2008 Relaxin signalling in primary cultures of human myometrial cells Mol. Hum. Reprod. 14 603 611 10.1093/molehr/gan051 18805799

[31] Liu J. Cai Y. Rahman K.U. Zhou Q. Liu G. Kang H. et al 2025 Rh-relaxin-2 attenuates oxidative stress and neuronal apoptosis via ERK-nNOS-NO pathway after germinal matrix hemorrhage in rats Fluids Barriers CNS 22 8 10.1186/s12987-024-00616-7 39815354 PMC11734463

[32] Safari Baesmat A. Bayrakdar B 2024 Emerging roles for the RXFP1 in myeloid series leukemia ijmsci 11 7161 7170 10.18535/ijmsci/v11i6.05

[33] Dschietzig T. Bartsch C. Greinwald M. Baumann G. Stangl K 2005 The pregnancy hormone relaxin binds to and activates the human glucocorticoid receptor Ann. N. Y. Acad. Sci. 1041 256 271 10.1196/annals.1282.039 15956716

[34] Bialek J. Piwonka M. Kawan F. Fornara P. Theil G 2021 Differential Expression of the Androgen Receptor, Splice Variants and Relaxin 2 in Renal Cancer Life (Basel). 11 731 10.3390/life11080731 34440475 PMC8402134

[35] Dschietzig T. Brecht A. Bartsch C. Baumann G. Stangl K. Alexiou K 2012 Relaxin improves TNF-α-induced endothelial dysfunction: the role of glucocorticoid receptor and phosphatidylinositol 3-kinase signalling Cardiovasc. Res. 95 97 107 10.1093/cvr/cvs149 22510373

[36] Cosen-Binker L.I. Binker M.G. Cosen R. Negri G. Tiscornia O 2006 Relaxin prevents the development of severe acute pancreatitis World J. Gastroenterol. 12 1558 1568 10.3748/wjg.v12.i10.1558 16570348 PMC4124288

[37] Dschietzig T. Bartsch C. Baumann G. Stangl K 2009a RXFP1-inactive relaxin activates human glucocorticoid receptor: further investigations into the relaxin–GR pathway Regul. Pept. 154 77 84 10.1016/j.regpep.2008.11.010 19101597

[38] Brecht A. Bartsch C. Baumann G. Stangl K. Dschietzig T 2011 Relaxin inhibits early steps in vascular inflammation Regul. Pept. 166 76 82 10.1016/j.regpep.2010.09.001 20851151

[39] Dschietzig T. Bartsch C. Wessler S. Baumann G. Stangl K 2009 Autoregulation of human relaxin-2 gene expression critically involves relaxin and glucocorticoid receptor binding to glucocorticoid response half-sites in the relaxin-2 promoter Regul. Pept. 155 163 173 10.1016/j.regpep.2009.03.001 19289144

[40] Horton J.S. Yamamoto S.Y. Bryant-Greenwood G.D 2011 Relaxin modulates proinflammatory cytokine secretion from human decidual macrophages1 Biol. Reprod. 85 788 797 10.1095/biolreprod.110.089201 21734258 PMC4480428

[41] Halls M.L. Bathgate R.A.D. Summers R.J 2007 Comparison of signaling pathways activated by the relaxin family peptide receptors, RXFP1 and RXFP2, using reporter genes J. Pharmacol. Exp. Ther. 320 281 290 10.1124/jpet.106.113225 17065365

[42] Kern A. Bryant-Greenwood G.D 2009 Characterization of relaxin receptor (RXFP1) desensitization and internalization in primary human decidual cells and RXFP1-transfected HEK293 cells Endocrinology 150 2419 2428 10.1210/en.2008-1385 19116340 PMC2671891

[43] Qin P. Pang Y. Hou W. Fu R. Zhang Y. Wang X. et al 2021 Integrated decoding hematopoiesis and leukemogenesis using single-cell sequencing and its medical implication Cell Discov. 7 2 10.1038/s41421-020-00223-4 33408321 PMC7788081

[44] Bani D. Maurizi M. Bigazzi M. Bani D. di D 1995 Original article: relaxin reduces the number of circulating platelets and depresses platelet release from megakaryocytes: studies in rats Platelets 6 330 335 10.3109/09537109509078467 21043760

[45] Bani D. Bigazzi M. Masini E. Bani G. Sacchi T.B 1995 Relaxin depresses platelet aggregation: in vitro studies on isolated human and rabbit platelets Lab. Invest. 73 709 716 7474945

[46] Bani D. Nistri S. Cinci L. Giannini L. Princivalle M. Elliott L. et al 2007 A novel, simple bioactivity assay for relaxin based on inhibition of platelet aggregation Regul. Pept. 144 10 16 10.1016/j.regpep.2007.05.004 17572516

[47] Grodzielski M. Cidlowski J.A 2023 Glucocorticoids regulate thrombopoiesis by remodeling the megakaryocyte transcriptome J. Thromb. Haemost 21 3207 3223 10.1016/j.jtha.2023.06.012 37336437 PMC10592358

[48] Moraes L.A. Paul-Clark M.J. Rickman A. Flower R.J. Goulding N.J. Perretti M 2005 Ligand-specific glucocorticoid receptor activation in human platelets Blood 106 4167 4175 10.1182/blood-2005-04-1723 16131566

[49] Nistri S. Cinci L. Perna A.M. Masini E. Mastroianni R. Bani D 2008 Relaxin induces mast cell inhibition and reduces ventricular arrhythmias in a swine model of acute myocardial infarction Pharmacol. Res. 57 43 48 10.1016/j.phrs.2007.11.001 18068999

[50] Masini E. Bani D. Bigazzi M. Mannaioni P.F. Bani-Sacchit T.E 1994 Effects of relaxin on mast cells. In vitro and in vivo studies in rats and guinea pigs J. Clin. Invest. 1974 1980 10.1172/JCI117549 7525651 PMC294619

[51] Li P. Zhao G. Chen F. Ding Y. Wang T. Liu S. et al 2020 Rh-relaxin-2 attenuates degranulation of mast cells by inhibiting NF-κB through PI3K-AKT/TNFAIP3 pathway in an experimental germinal matrix hemorrhage rat model J. Neuroinflammation 17 250 10.1186/s12974-020-01926-x 32859236 PMC7455905

[52] Masini E. Di Bello M.G. Bani D. Bigazzi M. Bani Sacchi T. Mannaioni P.F 1995 Relaxin inhibits histamine release from mast cells: involvement of nitric oxide production Inflamm. Res. 44 Suppl 1 S12 3 10.1007/BF01674372 8520977

[53] Masini E. Zagli G. Ndisang J.F. Solazzo M. Mannaioni P.F. Bani D 2002 Protective effect of relaxin in cardiac anaphylaxis: involvement of the nitric oxide pathway Br. J. Pharmacol. 137 337 344 10.1038/sj.bjp.0704879 12237253 PMC1573501

[54] Zhou J. Liu D. ‐F. Liu C. Kang Z. ‐M. Shen X. ‐H. Chen Y. ‐Z. et al 2008 Glucocorticoids inhibit degranulation of mast cells in allergic asthma via nongenomic mechanism Allergy 63 1177 1185 10.1111/j.1398-9995.2008.01725.x 18699934

[55] Uhlen M. Karlsson M.J. Zhong W. Tebani A. Pou C. Mikes J. et al 2019 A genome-wide transcriptomic analysis of protein-coding genes in human blood cells Science 366 eaax9198 10.1126/science.aax9198 31857451

[56] Karlsson M. Zhang C. Méar L. Zhong W. Digre A. Katona B. et al 2021 A single–cell type transcriptomics map of human tissues Sci. Adv. 7 10.1126/sciadv.abh2169 PMC8318366 34321199

[57] Monaco G. Lee B. Xu W. Mustafah S. Hwang Y.Y. Carré C. et al 2019 RNA-Seq Signatures Normalized by mRNA Abundance Allow Absolute Deconvolution of Human Immune Cell Types Cell Rep. 26 1627 1640 10.1016/j.celrep.2019.01.041 30726743 PMC6367568

[58] Jin H. Zhang C. Zwahlen M. Von Feilitzen K. Karlsson M. Shi M et al 2023 Systematic transcriptional analysis of human cell lines for gene expression landscape and tumor representation Nat. Commun. 14 5417 10.1038/s41467-023-41132-w 37669926 PMC10480497

[59] Goldsmith L.T. Weiss G 2009 Relaxin in human pregnancy Ann. N. Y. Acad. Sci. 1160 130 135 10.1111/j.1749-6632.2008.03800.x 19416173 PMC3856209

[60] Gao X.-M. Su Y. Moore S. Han L.-P. Kiriazis H. Lu Q. et al 2019 Relaxin mitigates microvascular damage and inflammation following cardiac ischemia–reperfusion Basic Res. Cardiol. 114 10.1007/s00395-019-0739-9 31218471

[61] Bolton A.E. Pockley A.G. Clough K.J. Mowles E.A. Stoker R.J. Westwood O.M. et al 1987 Identification of placental protein 14 as an immunosuppressive factor in human reproduction Lancet 1 593 595 10.1016/s0140-6736(87)90235-2 2881133

[62] Seppälä M. Koistinen H. Koistinen R. Chiu P.C.N. Yeung W.S.B Glycodelin: a lipocalin with diverse glycoform-dependent actions https://www.ncbi.nlm.nih.gov/books/NBK6332

[63] Tseng L. Zhu H.H. Mazella J. Koistinen H. Seppälä M 1999 Relaxin stimulates glycodelin mRNA and protein concentrations in human endometrial glandular epithelial cells Mol. Hum. Reprod. 5 372 375 10.1093/molehr/5.4.372 10321810

[64] Stewart D.R. Erikson M.S. Erikson M.E. Nakajima S.T. Overstreet J.W. Lasley B.L. et al 1997 The Role of Relaxin in Glycodelin Secretion The Journal of Clinical Endocrinology & Metabolism 82 839 846 10.1210/jcem.82.3.3839 9062493

[65] Meola J. Dentillo D.B. Rosa e Silva J.C. Ferriani R.A. Veiga L.C. Paro de Paz C.C. et al 2009 Glycodelin expression in the endometrium of healthy women and in the eutopic and ectopic endometrium of women with endometriosis Fertil. Steril. 91 1676 1680 10.1016/j.fertnstert.2008.02.158 18402941

[66] Halttunen M. Kämäräinen B., K. Koistinen H 2000 Glycodelin: a reproduction-related lipocalin Biochim. Biophys. Acta 149 156 10.1016/s0167-4838(00)00158-8 11058757

[67] Taylor R.N. Ois J.-F. Vaisse C. Vigne J.-L. Ryan I. Hornung D et al 1998 Promegestone (R5020) and Mifepristone (RU486) Both Function as Progestational Agonists of Human Glycodelin Gene Expression in Isolated Human Epithelial Cells https://academic.oup.com/jcem/article/83/11/4006/2865647 10.1210/jcem.83.11.5214 9814484

[68] Schoch G.A. D’Arcy B. Stihle M. Burger D. Bär D. Benz J. et al 2010 Molecular switch in the glucocorticoid receptor: active and passive antagonist conformations J. Mol. Biol. 395 568 577 10.1016/j.jmb.2009.11.011 19913032

[69] Cooper D.N.W 2002 Galectinomics: finding themes in complexity Biochimica et Biophysica Acta (BBA) - General Subjects 1572 209 231 10.1016/S0304-4165(02)00310-0 12223271

[70] Aragón-Herrera A. Couselo-Seijas M. Feijóo-Bandín S. Anido-Varela L. Moraña-Fernández S. Tarazón E. et al 2022 Relaxin-2 plasma levels in atrial fibrillation are linked to inflammation and oxidative stress markers Sci. Rep. 12 22287 10.1038/s41598-022-26836-1 36566255 PMC9789945

[71] Masini E. Nistri S. Vannacci A. Bani Sacchi T. Novelli A. Bani D 2004 Relaxin inhibits the activation of human neutrophils: involvement of the nitric oxide pathway Endocrinology 145 1106 1112 10.1210/en.2003-0833 14630720

[72] Nistri S. Chiappini L. Sassoli C. Bani D 2003 Relaxin inhibits lipopolysaccharide-induced adhesion of neutrophils to coronary endothelial cells by a nitric oxide-mediated mechanism FASEB J. 17 2109 2111 10.1096/fj.03-0216fje 14500542

[73] Bhagavathi Perumal M. Dhanasekaran S 2014 Relaxin: A missing link in the pathomechanisms of Systemic Lupus Erythematosus? Mod. Rheumatol. 24 547 551 10.3109/14397595.2013.844297 24251992

[74] Bani D. Masini E. Bello M.G. Bigazzi M. Sacchi T.B 1998 Relaxin protects against myocardial injury caused by ischemia and reperfusion in rat heart Am. J. Pathol. 152 1367 1376 9588905 PMC1858569

[75] Lin W. Chen H. Chen X. Guo C 2024 The roles of neutrophil-derived myeloperoxidase (MPO) in diseases: the new progress Antioxidants (Basel). 13 132 10.3390/antiox13010132 38275657 PMC10812636

[76] Filep J.G. Delalandre A. Payette Y. Földes-Filep E 1997 Glucocorticoid receptor regulates expression of L-Selectin and CD11/CD18 on Human Neutrophils Circulation 96 295 301 10.1161/01.CIR.96.1.295 9236448

[77] Hill G.E. Alonso A. Thiele G.M. Robbins R.A Glucocorticoids blunt neutrophil CD11b surface glycoprotein upregulation during cardiopulmonary bypass in humans Anesth. Analg. 23 27 10.1213/00000539-199407000-00006 7912043

[78] Bani D. Baronti R. Vannacci A. Bigazzi M. Sacchi T.B. Mannaioni P.F et al Inhibitory effects of relaxin on human basophils activated by stimulation of the Fc epsilon receptor. The role of nitric oxide Int. Immunopharmacol. 1195 1204 10.1016/s1567-5769(02)00079-6 12349956

[79] Pellefigues C. Mehta P. Chappell S. Yumnam B. Old S. Camberis M. et al 2021 Diverse innate stimuli activate basophils through pathways involving Syk and IκB kinases Proc. Natl. Acad. Sci. U.S.A. 118 e2019524118 10.1073/pnas.2019524118 33727419 PMC8000355

[80] Yamagata S. Tomita K. Sano H. Itoh Y. Fukai Y. Okimoto N. et al 2012 Non-genomic inhibitory effect of glucocorticoids on activated peripheral blood basophils through suppression of lipid raft formation Clin. Exp. Immunol. 170 86 93 10.1111/j.1365-2249.2012.04636.x 22943204 PMC3444720

[81] Yuan S. Guo D. Liang X. Zhang L. Zhang Q. Xie D 2023 Relaxin in fibrotic ligament diseases: Its regulatory role and mechanism Front. Cell Dev. Biol. 11 1131481 10.3389/fcell.2023.1131481 37123405 PMC10134402

[82] Luque E.H. Muñoz de Toro M.M. Ramos J.G. Rodriguez H.A. Sherwood O.D 1998 Role of relaxin and estrogen in the control of eosinophilic invasion and collagen remodeling in rat cervical tissue at term Biol. Reprod. 59 795 800 10.1095/biolreprod59.4.795 9746727

[83] Kristiansson P. Holding C. Hughes S. Haynes D 2005 Does human relaxin-2 affect peripheral blood mononuclear cells to increase inflammatory mediators in pathologic bone loss? In: Annals of the New York Academy of Sciences Ann. N. Y. Acad. Sci. 1041 317 319 10.1196/annals.1282.050 15956727

[84] Figueiredo K.A. Mui A.L. Nelson C.C. Cox M.E 2006 Relaxin stimulates leukocyte adhesion and migration through a relaxin receptor LGR7-dependent mechanism J. Biol. Chem. 281 3030 3039 10.1074/jbc.M506665200 16303766

[85] Piccinni M.P. Bani D. Beloni L. Manuelli C. Mavilia C. Vocioni F. et al 1999 Relaxin favors the development of activated human T cells into Th1-like effectors Eur. J. Immunol. 29 2241 2247 10.1002/(SICI)1521-4141(199907)29:07<2241::AID-IMMU2241>3.0.CO;2-E 10427987

[86] Bagazzi M 2005 Use of relaxin for stimulating the development of activated human T cells into TH1-like effectors US20050180968A1

[87] Taves M.D. Ashwell J.D 2021 Glucocorticoids in T cell development, differentiation and function Nat. Rev. Immunol. 21 233 243 10.1038/s41577-020-00464-0 33149283

[88] Wolf V.L. Phillips T.L. Taylor E.B. Sasser J.M. Ryan M.J 2019 Human recombinant relaxin-2 does not attenuate hypertension or renal injury but exacerbates vascular dysfunction in a female mouse model of SLE Am. J. Physiol. Heart Circ. Physiol. 317 H234 H242 10.1152/ajpheart.00174.2019 31125285 PMC6732478

[89] Anand-Ivell R. Heng K. Bartsch O. Ivell R 2007 Relaxin signalling in THP-1 cells uses a novel phosphotyrosine-dependent pathway Mol. Cell. Endocrinol. 272 1 13 10.1016/j.mce.2007.04.001 17509748

[90] Nguyen B.T. Yang L. Sanborn B.M. Dessauer C.W 2003 Phosphoinositide 3-kinase activity is required for biphasic stimulation of cyclic adenosine 3’,5’-monophosphate by relaxin Mol. Endocrinol. 17 1075 1084 10.1210/me.2002-0284 12595573

[91] Beiert T. Knappe V. Tiyerili V. Stöckigt F. Effelsberg V. Linhart M et al 2018 Chronic lower-dose relaxin administration protects from arrhythmia in experimental myocardial infarction due to anti-inflammatory and anti-fibrotic properties Int. J. Cardiol. 250 21 28 10.1016/j.ijcard.2017.09.017 29169754

[92] Zhou X. Liu Y. Hu M. Wang M. Liu X. Huang L 2021 Relaxin gene delivery modulates macrophages to resolve cancer fibrosis and synergizes with immune checkpoint blockade therapy Sci. Adv. 7 eabb6596 10.1126/sciadv.abb6596 33597232 PMC7888957

[93] Kageyama S. Nakamura K. Ke B. Busuttil R.W. Kupiec-Weglinski J.W 2018 Serelaxin induces Notch1 signaling and alleviates hepatocellular damage in orthotopic liver transplantation Am. J. Transplant. 18 1755 1763 10.1111/ajt.14706 29464890 PMC6035063

[4] Young L. Katrib A. Cuello C. Vollmer-Conna U. Bertouch J.V. Roberts-Thomson P.J. et al 2001 Effects of intraarticular glucocorticoids on macrophage infiltration and mediators of joint damage in osteoarthritis synovial membranes: Findings in a double-blind, placebo-controlled study Arthritis & Rheumatism 44 343 350 10.1002/1529-0131(200102)44:2<343::AID-ANR52>3.0.CO;2-Q 11229465

[95] Figueiredo K.A. Rossi G. Cox M.E 2009 Relaxin promotes clustering, migration, and activation states of mononuclear myelocytic cells: Implications of leukocyte responsiveness to tumor-derived relaxin In Annals of the New York Academy of Sciences.. Blackwell Publishing Inc pp 353 360 10.1111/j.1749-6632.2009.03843.x 19416219

[96] Binder C. Chuang E. Habla C. Bleckmann A. Schulz M. Bathgate R. et al 2014 Relaxins enhance growth of spontaneous murine breast cancers as well as metastatic colonization of the brain Clin. Exp. Metastasis 31 57 65 10.1007/s10585-013-9609-2 23963762 PMC3892110

[97] Bartsch O. Bartlick B. Ivell R 2001 Relaxin signalling links tyrosine phosphorylation to phosphodiesterase and adenylyl cyclase activity Mol. Hum. Reprod. 7 799 809 10.1093/molehr/7.9.799 11517286

[98] Zhang Q. Liu S.H. Erikson M. Lewis M. Unemori D.E 2002 Relaxin activates the MAP kinase pathway in human endometrial stromal cells J. Cell. Biochem. 85 536 544 10.1002/jcb.10150 11967993

[99] Arancibia S. Benítez D. Núñez L.E. Jewell C.M. Langjahr P. Candia E. et al 2011 Phosphatidylinositol 3-kinase interacts with the glucocorticoid receptor upon TLR2 activation J. Cell. Mol. Med. 15 339 349 10.1111/j.1582-4934.2009.00958.x 19874421 PMC3822800

[100] Unemori E.N. Lewis M. Constant J. Arnold G. Grove B.H. Normand J. et al 2000 Relaxin induces vascular endothelial growth factor expression and angiogenesis selectively at wound sites Wound Repair Regen. 8 361 370 10.1111/j.1524-475x.2000.00361.x 11186125

[101] Itaya H. Imaizumi T. Yoshida H. Koyama M. Suzuki S. Satoh K 2001 Expression of vascular endothelial growth factor in human monocyte/macrophages stimulated with lipopolysaccharide Thromb. Haemost. 85 171 176 10.1055/s-0037-1612921 11204570

[102] Ho T.Y. Bagnell C.A 2005 Relaxin induces matrix metalloproteinase-9 through activation of nuclear factor kappa B in human THP-1 cells Ann. N. Y. Acad. Sci. 1041 314 316 10.1196/annals.1282.049 15956726

[103] Cruz-Topete D. Cidlowski J.A 2015 One hormone, two actions: anti- and pro-inflammatory effects of glucocorticoids Neuroimmunomodulation 22 20 32 10.1159/000362724 25227506 PMC4243162

[104] Smoak K.A. Cidlowski J.A 2004 Mechanisms of glucocorticoid receptor signaling during inflammation Mech. Ageing Dev. 125 697 706 10.1016/j.mad.2004.06.010 15541765

[105] Haggerty D.F. Spector E.B. Lynch M. Kern R. Frank L.B. Cederbaum S.D 1982 Regulation of glucocorticoids of arginase and argininosuccinate synthetase in cultured rat hepatoma cells J. Biol. Chem. 257 2246 2253 10.1016/s0021-9258(18)34913-5 7061421

[106] Ouyang S. Liu C. Xiao J. Chen X. Lui A.C. Li X 2020 Targeting IL-17A/glucocorticoid synergy to CSF3 expression in neutrophilic airway diseases JCI Insight 5 e132836 10.1172/jci.insight.132836 32051346 PMC7098787

[107] Strizova Z. Benesova I. Bartolini R. Novysedlak R. Cecrdlova E. Foley L.K. et al 2023 M1/M2 macrophages and their overlaps – myth or reality? Clin. Sci. 137 1067 1093 10.1042/CS20220531 PMC10407193 37530555

[108] Chen L. Sha M.-L. Li D. Zhu Y.-P. Wang X.-J. Jiang C.-Y. et al 2017 Relaxin abrogates renal interstitial fibrosis by regulating macrophage polarization via inhibition of Toll-like receptor 4 signaling Oncotarget 8 21044 21053 10.18632/oncotarget.15483 28416741 PMC5400564

[109] Xie Y. Tolmeijer S. Oskam J.M. Tonkens T. Meijer A.H. Schaaf M.J.M 2019 Glucocorticoids inhibit macrophage differentiation towards a pro-inflammatory phenotype upon wounding without affecting their migration Dis. Model. Mech. 12 dmm037887 10.1242/dmm.037887 31072958 PMC6550045

[110] Ng Kuet Leong N. Brombacher F. Dalpke A.H. Weitnauer M 2017 Crosstalk between glucocorticoids and IL-4 modulates Ym1 expression in alternatively activated myeloid cells Immunobiology 222 759 767 10.1016/j.imbio.2017.02.003 28209270

[111] Gupte R. Muse G.W. Chinenov Y. Adelman K. Rogatsky I 2013 Glucocorticoid receptor represses proinflammatory genes at distinct steps of the transcription cycle Proc. Natl. Acad. Sci. U.S.A. 110 14616 14621 10.1073/pnas.1309898110 23950223 PMC3767553

[112] Desgeorges T. Caratti G. Mounier R. Tuckermann J. Chazaud B 2019 Glucocorticoids Shape Macrophage Phenotype for Tissue Repair Front. Immunol. 10 1591 10.3389/fimmu.2019.01591 31354730 PMC6632423

[113] Peñuelas I. Encío I.J. López-Moratalla N. Santiago E 1998 cAMP activates transcription of the human glucocorticoid receptor gene promoter J. Steroid Biochem. Mol. Biol. 89 94 10.1016/s0960-0760(98)00097-1 9877208

[114] Young-min P. In-deok J. Seung-jun L 2017 Composition for inducing maturation of antigen-presenting cells comprising relaxin KR101739305B1

[115] Bauer A. Tronche F. Wessely O. Kellendonk C. Reichardt H.M. Steinlein P. et al 1999 The glucocorticoid receptor is required for stress erythropoiesis Genes Dev. 13 2996 3002 10.1101/gad.13.22.2996 10580006 PMC317156

[116] Ronchetti S. Ricci E. Migliorati G. Gentili M. Riccardi C 2018 How Glucocorticoids Affect the Neutrophil Life Int. J. Mol. Sci. 19 4090 10.3390/ijms19124090 30563002 PMC6321245

[117] Zen M. Canova M. Campana C. Bettio S. Nalotto L. Rampudda M. et al 2011 The kaleidoscope of glucorticoid effects on immune system Autoimmun. Rev. 10 305 310 10.1016/j.autrev.2010.11.009 21224015

[118] Eddleston J. Herschbach J. Wagelie-Steffen A.L. Christiansen S.C. Zuraw B.L 2007 The anti-inflammatory effect of glucocorticoids is mediated by glucocorticoid-induced leucine zipper in epithelial cells Journal of Allergy and Clinical Immunology 119 115 122 10.1016/j.jaci.2006.08.027 17208592

[119] Steer J.H. Kroeger K.M. Abraham L.J. Joyce D.A 2000 Glucocorticoids suppress tumor necrosis factor-alpha expression by human monocytic THP-1 cells by suppressing transactivation through adjacent NF-kappa B and c-Jun-activating transcription factor-2 binding sites in the promoter J. Biol. Chem. 275 18432 18440 10.1074/jbc.M906304199 10748079

[120] Scheinman R.I. Cogswell P.C. Lofquist A.K. Baldwin A.S. Jr 1995 Role of transcriptional activation of I kappa B alpha in mediation of immunosuppression by glucocorticoids Science 270 283 286 10.1126/science.270.5234.283 7569975

[121] Liu B. Goodwin J.E 2020 The effect of glucocorticoids on angiogenesis in the treatment of solid tumors Journal of cellular signaling 42 49 10.33696/Signaling.1.011 32728672 PMC7388649

[122] Wang Z. Chen W. Kono E. Dang T. Garabedian M.J 2007 Modulation of glucocorticoid receptor phosphorylation and transcriptional activity by a C-terminal-associated protein phosphatase Mol. Endocrinol. 21 625 634 10.1210/me.2005-0338 17185395

[123] Galliher-Beckley A.J. Cidlowski J.A 2009 Emerging roles of glucocorticoid receptor phosphorylation in modulating glucocorticoid hormone action in health and disease IUBMB Life 61 979 986 10.1002/iub.245 19787703

[124] Sato N. Takahashi W. Hirayama A. Ajioka M. Takahashi N. Okishige K. et al 2015 Multicenter, randomized, double-blinded, placebo-controlled phase ii study of serelaxin in japanese patients with acute heart failure Circ. J. 79 1237 1247 10.1253/circj.CJ-15-0227 25912697

[125] Teerlink J.R. Cotter G. Davison B.A. Felker G.M. Filippatos G. Greenberg B.H et al 2013 Serelaxin, recombinant human relaxin-2, for treatment of acute heart failure (RELAX-AHF): a randomised, placebo-controlled trial Lancet 381 29 39 10.1016/S0140-6736(12)61855-8 23141816

[126] Dschietzig T. Teichman S. Unemori E. Wood S. Boehmer J. Richter C. et al 2009 Intravenous recombinant human relaxin in compensated heart failure: a safety, tolerability, and pharmacodynamic trial J. Card. Fail. 15 182 190 10.1016/j.cardfail.2009.01.008 19327619

[127] Snowdon V.K. Lachlan N.J. Hoy A.M. Hadoke P.W.F. Semple S.I. Patel D. et al 2017 Serelaxin as a potential treatment for renal dysfunction in cirrhosis: Preclinical evaluation and results of a randomized phase 2 trial PLoS Med. 14 e1002248 10.1371/journal.pmed.1002248 28245243 PMC5330452

[128] Weiss G. Teichman S. Stewart D. Nader D. Wood S. Breining P. et al 2016 Recombinant human relaxin versus placebo for cervical ripening: a double-blind randomised trial in pregnant women scheduled for induction of labour BMC Pregnancy Childbirth 16 260 10.1186/s12884-016-1046-1 27596360 PMC5011832

[129] Seibold J.R. Clements P.J. Furst D.E. Mayes M.D. McCloskey D.A. Moreland L.W. et al 1998 Safety and pharmacokinetics of recombinant human relaxin in systemic sclerosis J. Rheumatol. 25 302 307 9489823

[130] Azam A. Gallais Y. Mallart S. Illiano S. Duclos O. Prades C. et al 2019 Healthy Donors Exhibit a CD4 T Cell Repertoire Specific to the Immunogenic Human Hormone H2-Relaxin before Injection J. Immunol. 202 3507 3513 10.4049/jimmunol.1800856 31101669

